# Recent Advances in α-Diimine Nickel Complexes for the Synthesis of Polyethylene Elastomers via Chain Walking

**DOI:** 10.3390/polym18161933

**Published:** 2026-08-07

**Authors:** Tian Liu, Rong Gao, Qingqiang Gou, Randi Zhang, Jingshuang Yang, Jingjing Lai, Qiang Yue, Ying Wang

**Affiliations:** Department of Polyethylene, SINOPEC (Beijing) Research Institute of Chemical Industry Co. Ltd., Beijing 100013, China

**Keywords:** α-diimine nickel(II) complex, ethylene polymerization, chain-walking mechanism, the electronic and steric effects, performance adjustment

## Abstract

Polyolefin elastomers (POEs) produced by ethylene/α-olefin copolymerization are indispensable for high-end applications such as photovoltaic encapsulation, precision microelectronic protection, advanced foamed footwear, and automotive lightweight components. α-Diimine nickel catalysts that operate through a chain-walking mechanism convert ethylene as the sole feedstock into highly branched polyethylene elastomers, eliminating the need for expensive comonomers. This review systematically analyzes α-diimine nickel complexes developed in recent years via modulation of ligand steric hindrance, electronic effects, and backbone rigidity, and provides a quantitative comparison of their catalytic performance. The compiled data reveal that the catalytic activities span 10^4^–10^7^ g PE (mol Ni)^−1^ h^−1^, the molecular weights range from 10^4^ to 10^6^ g mol^−1^, and the polydispersity indices (PDIs) can be tuned between 1.2 and 29.3, affording polyethylenes with branching densities from 2 to over 186 branches per 1000 carbons. These structural parameters directly govern the mechanical flexibility, elastic recovery, thermal properties, and processability in injection molding, foam extrusion, and film blowing, thus dictating the materials’ suitability for the aforementioned high-value applications. By establishing clear structure–performance relationships, this review offers forward-looking guidance for the industrial scale-up and catalyst design of polyethylene elastomers produced exclusively from ethylene.

## 1. Introduction

Polyolefin materials, primarily manufactured from ethylene, propylene, and long-chain α-olefins via coordination or free-radical polymerization, have been extensively utilized across consumer packaging, medical devices, and industrial equipment, serving as critical alternatives to metals, wood, and glass [1]. Among them, thermoplastic polyolefin elastomers (POEs) produced by ethylene/α-olefin copolymerization have emerged as technology-intensive, high-value-added materials owing to their outstanding flexibility, aging resistance, and elastic recovery, making them indispensable for photovoltaic encapsulation, precision microelectronic protection, advanced foamed footwear, and automotive lightweight components [2,3]. However, the global supply of long-chain α-olefins such as 1-hexene and 1-octene remains structurally constrained, and major petrochemical corporations have established formidable patent and process barriers around high-temperature solution polymerization [4], severely limiting the large-scale independent industrialization of POEs for the vast majority of R&D entities.

To reduce dependence on costly α-olefin comonomers and circumvent existing intellectual property barriers, late transition metal catalysts that convert ethylene as the sole feedstock into branched polyethylene elastomers have attracted intense interest. Nickel-catalyzed ethylene oligomerization and polymerization date back to the Shell Higher Olefin Process (SHOP) [5], while the field was revolutionized by Brookhart’s discovery of α-diimine nickel and palladium complexes, which operate via a unique “chain-walking” mechanism and generate highly branched to hyperbranched polyethylene topologies from ethylene alone [6,7]. Since that seminal work, a wide array of nickel and palladium catalysts bearing various bidentate ligands—including phosphine-sulfonate, phosphine-phenolate, salicylaldimine, and N-heterocyclic carbene-based systems—have been developed and shown to produce polyolefin elastomers with diverse branching architectures [8,9]. Although palladium catalysts exhibit remarkable functional group tolerance, nickel-based systems are far more attractive for industrial application because of their higher catalytic activity, substantially lower metal cost, and the ability to achieve a broader range of polymer molecular weights. Within the nickel family, α-diimine nickel(II) complexes stand out for their exceptional structural tunability: the steric and electronic properties of the diimine ligand can be precisely modulated to control the relative rates of chain walking versus monomer insertion, thereby providing comprehensive regulation over molecular weight, polydispersity, branching density, and branch-type distribution [10,11]. This full-spectrum regulatory capability—spanning from lubricant base oil additives to film resins and ultrahigh-molecular-weight engineering plastics—has established a solid theoretical and experimental foundation for the direct production of commercially viable polyethylene elastomers from ethylene as the sole monomer.

Given the unparalleled versatility and the rapid recent advances in ligand design for α-diimine nickel catalysts, the present review focuses exclusively on these systems. We systematically analyze novel α-diimine nickel complexes developed in recent years through strategies including modulation of ligand steric hindrance, fine-tuning of electronic effects, and expansion of backbone rigidity, and we thoroughly explore the intrinsic relationship between catalyst molecular structure and polyethylene elastomer performance. By establishing clear structure–performance correlations and comparative performance data, this review aims to provide forward-looking guidance for the industrial scale-up and formulation design of ethylene-based polyethylene elastomers.

## 2. In-Depth Analysis of the Chain-Walking Mechanism

**Scheme 1:**
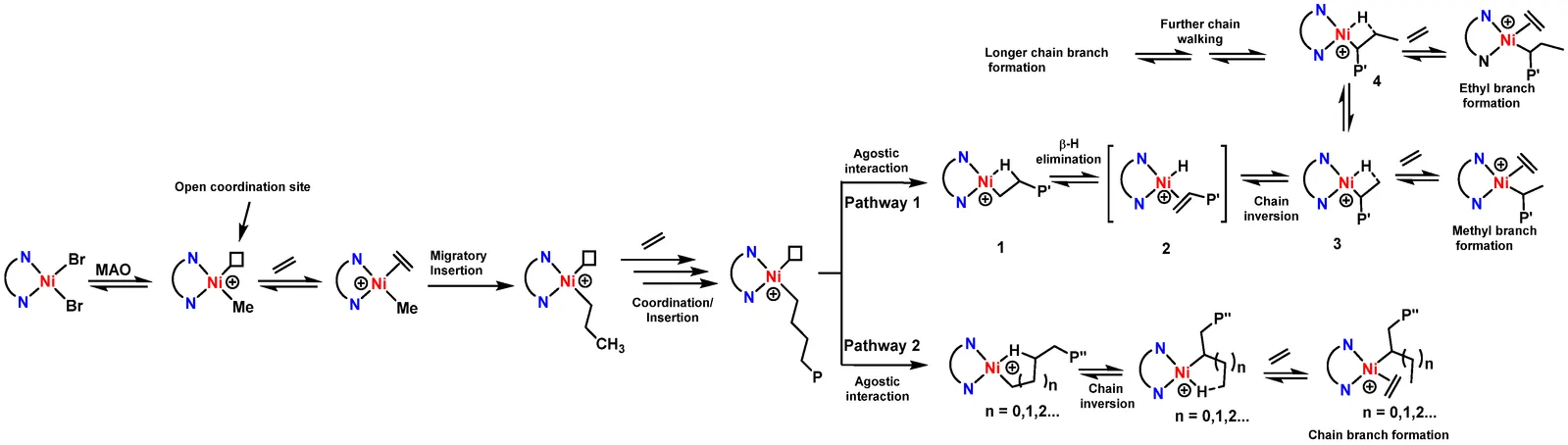
The “Chain Walking” mechanism of the branched polyethylene formation with *α*-diimine nickel(II) complex.

The fundamental reason α-diimine nickel complexes can transform ethylene into elastomers possessing complex network architectures-in sharp contrast to other transition-metal-catalyzed polymerization systems-lies in their unique “chain-walking” microscopic polymerization kinetics. The generally accepted chain-walking mechanism is supported by extensive experimental and computational studies [7,12,13,14,15]. During chain propagation, the electrophilic nickel center engages in subtle interactions with hydrogen atoms located at different positions along the growing polymer chain, such as β-H, γ-H, ω-H, and even higher-order interactions involving more remote hydrogen atoms; subsequently, the polymer chain rotates 180° about the M-η^2^-olefin bond, and the hydrogen atom ultimately reattaches to the terminal carbon of the chain [16,17,18]. These interactions can give rise to methyl, ethyl, propyl, or even longer branches, depending on which hydrogen atom is initially abstracted (Scheme 1).

Furthermore, to rationalize the peculiar observation that only methyl branches and long-chain branches are detected in certain catalytic systems, Pei et al. proposed a more in-depth mechanistic explanation based on experimental results and further computational studies [19]. They argued that the N-aryl substituents construct a significantly sterically hindered environment around the nickel center, which restricts ethylene insertion exclusively to primary or secondary alkyl nickel active species located at the α-position of a methyl branch. This stringent steric constraint completely blocks the formation of medium-length branches such as ethyl, propyl, and butyl through the conventional chain-walking pathway; nevertheless, the nickel center, by virtue of its high migratory aptitude, can still undergo multiple successive hopping walks along the polymer backbone already bearing methyl branches. Once the metal center migrates back to the methyl terminus, the active species reverts to a primary alkyl nickel species, thereby re-satisfying the steric requirement for ethylene insertion and facilitating the initiation and further growth of new long-chain branches.

Beyond explaining the chain walking mechanism, theoretical calculations have played an important role in understanding monomer insertion and chain transfer. DFT calculations have consistently identified ethylene insertion into the nickel–alkyl bond as the rate-determining step in the catalytic cycle. They quantify how the free-energy barrier for insertion is modulated by ligand architecture: bulky ortho substituents create a congested axial site that destabilizes the resting state and lowers the insertion barrier, while strongly electron-withdrawing substituents enhance the electrophilicity of the metal center, thereby facilitating ethylene coordination and subsequent migratory insertion. Regarding chain transfer, DFT analyses have identified β-hydride transfer to monomer as the dominant chain-transfer pathway and have determined its free-energy barrier relative to that of insertion. The difference between these two barriers effectively governs the polymer molecular weight. Calculations show that increasing ligand steric bulk raises the transfer barrier more substantially than the insertion barrier, thereby suppressing chain transfer and yielding higher molecular weights. Some studies have also examined chain transfer to alkylaluminum cocatalysts, providing a more complete picture of termination events [7].

Based on the above findings, we hypothesize that both the ligand backbone architecture and the type of N-aryl substituents can modulate the electronic [20,21] and steric [22,23] effects exerted by the ligand framework, thereby altering the coordination environment of the metal center, tuning the chain-walking process accordingly, and ultimately influencing the polymer microstructure [7]. In particular, the incorporation of electron-withdrawing substituents into the complex significantly enhances the electrophilicity of the metal center, which favors chain propagation and promotes the formation of high-molecular-weight polyethylene [20]. In addition, polymerization conditions-including temperature, ethylene feed pressure, and cocatalyst concentration-can also directly affect the chain-walking process and therefore the topological architecture of the resulting polymer, enabling the synthesis of products spanning from hyperbranched amorphous segments to linear semicrystalline segments from ethylene as the sole monomer [7,24,25]. For instance, ethylene pressure directly governs the rate at which the metal center captures ethylene monomer. Under high ethylene pressure, the monomer capture rate by the nickel center is rapid; consequently, the average walking distance of the nickel center along the growing chain is relatively short, yielding topologies that are predominantly relatively linear with moderate branching. In contrast, under low ethylene pressure, monomer capture is slow relative to the chain-walking process, allowing the nickel center to undergo long-distance migration along the polyethylene chain prior to ethylene insertion. This promotes repeated branching on existing branches and results in hyperbranched topologies [7].

## 3. Structural Evolution of α-Diimine Nickel(II) Complexes

**Figure 1:**
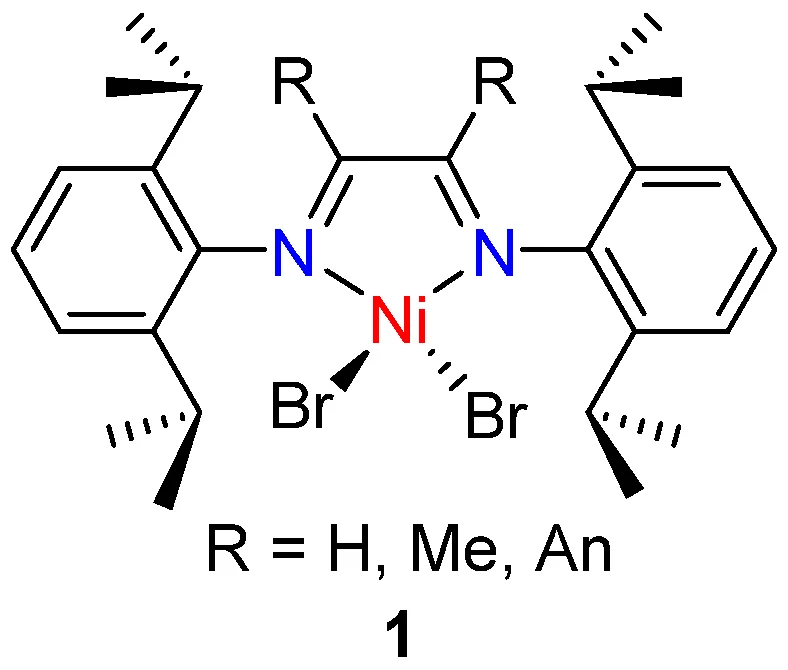
Brookhart’s proposed α-diimine nickel(II) complex **1**.

Building upon the classic Brookhart-type complex backbone (Figure 1), researchers have undertaken further innovative structural designs of α-diimine nickel(II) complexes. It has been found that altering the ligand backbone, introducing different N-aryl substituents, or constructing bimetallic cooperative systems can each significantly influence catalytic activity and polymer properties. To systematically elucidate these structure-property relationships, this review presents an in-depth discussion from three perspectives: steric effects, electronic effects, and bimetallic synergistic effects.

### 3.1. Steric and Electronic Modulation by N-Aryl Substituents

Within the molecular architecture of α-diimine nickel(II) complexes, the substituents on the N-aryl rings-particularly those at the ortho positions-impose a pronounced steric shielding effect around the active metal center, thereby directly influencing the energy barrier for monomer insertion and the propensity for chain transfer reactions.

#### 3.1.1. Exploration of Fluorine-Substituted Nickel Complex Structures

**Figure 2:**
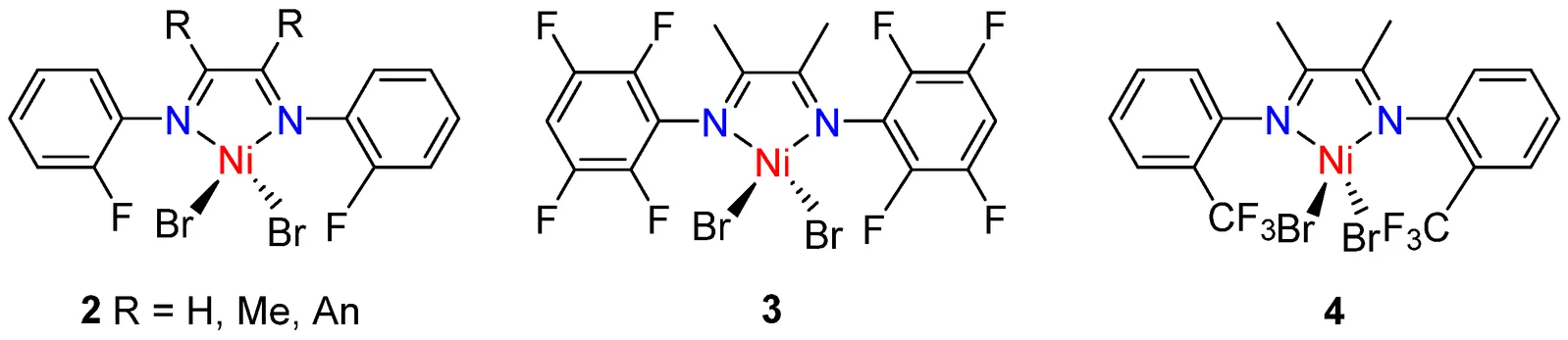
Fluorine-substituted *α*-diimine nickel(II) complexes **2**–**4**.

Among various substituents, the fluorine atom, by virtue of its exceedingly small van der Waals radius and distinctly weak electron-withdrawing character, constitutes an ideal probe for tuning the electronic properties of the central metal. Researchers have speculated that weak interactions may readily develop between β-H atoms of the growing polymer chain and *ortho*-fluorine atoms on the N-aryl rings, thereby effectively suppressing β-H elimination [26]. For instance, complex **2** (Figure 2), designed and synthesized by Ahmadjo et al. [27], incorporates fluorine atoms at the ortho positions of the N-aryl rings; while retaining an open coordination environment, this complex sustains a moderate catalytic activity of 1.40 × 10^4^ g⋅mol^−1^⋅h^−1^ (30 °C, 30 min). Moreover, the resulting polyethylene attains a number-average molecular weight (*M_n_*) of 2.48 × 10^5^ g·mol^−1^ and a crystallinity as high as 58%, exhibiting the hallmarks of a semicrystalline polymer. Nevertheless, not all fluorine-containing modifications prove effective: complexes **3** and **4** [28], which bear polyfluoro substituents or trifluoromethyl groups, catalyze only the formation of oily oligomers with degrees of polymerization ranging from 6 to 9.

**Figure 3:**
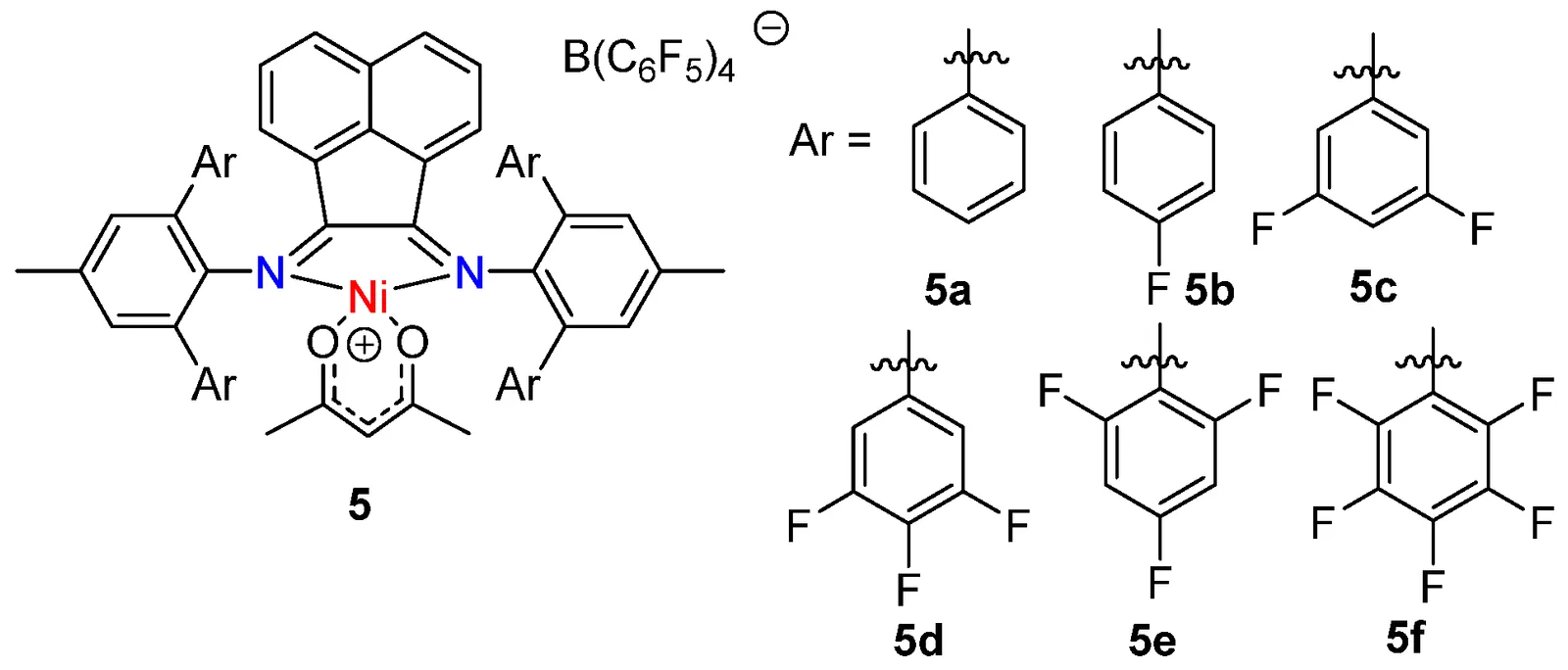
Fluorine-substituted *α*-diimine nickel(II) complex **5**.

Hu et al. [29] conducted a systematic investigation into the effect of fluorine substitution position (complex **5** series, Figure 3) and revealed the decisive role of positional effects: when the fluorine atoms are situated at the ortho positions (e.g., complexes **5e** and **5f**), the synergistic combination of steric hindrance and electronic effects effectively suppresses β-H elimination, yielding highly linear high-density polyethylene with a branching density as low as 1 branch per 1000 carbon atoms and a molecular weight (*M_w_*) of 8.70 × 10^3^ g·mol^−1^. In contrast, relocating the fluorine atoms to the meta or para positions (e.g., **5b**, **5c**, **5d**) removes the proximate shielding of the metal center, markedly enhancing chain walking and resulting in products with *M_w_* values in the range of (20–50) × 10^3^ g·mol^−1^ and a branching density up to 80 branches per 1000 carbon atoms, thereby reducing the crystallinity of the resulting polymer and endowing it with soft, elastomeric characteristics. It is worth noting that, although the fully fluorinated complex **5f** contains five fluorine atoms, both its catalytic activity and the molecular weight of the resulting polymer are inferior to those of **5e**, which bears only *ortho*-fluorine substitution, indicating that an excessively strong electron-withdrawing effect may adversely affect catalytic performance.

#### 3.1.2. Exploration of Nickel Complexes Bearing Flexible Cycloalkyl *Ortho*-Substituents

**Figure 4:**
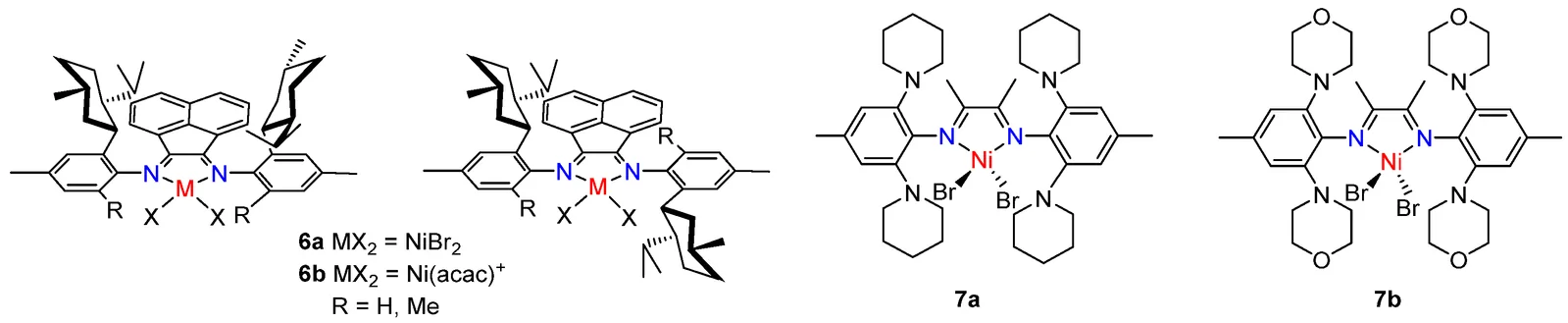
*Ortho*-menthyl and nitrogen heterocyclic group-modified *α*-diimine nickel(II) complexes **6**–**7**.

To probe the protective mechanism exerted by dynamic steric hindrance on the metal center, researchers introduced sterically tunable flexible cycloalkyl groups at the ortho positions of the N-aryl rings. Among these efforts, Zhai et al. synthesized a family of complexes **6** (Figure 4) bearing *ortho*-menthyl substituents [30]; these complexes exhibit pronounced syn/anti isomerism, and the two isomers display drastically different catalytic behaviors in ethylene polymerization. Upon activation with Et_2_AlCl, complex **6a**, which favors a syn conformation, exhibited exceptionally high catalytic activity, reaching (2.5–6.6) × 10^6^ g·mol^−1^·h^−1^ (22 °C, 15 min, 0.1 MPa). In contrast, the anti-configured complex **6b** not only showed lower activity, but also produced a polymer with significantly lower branching density and molecular weight than its syn counterpart, thus suggesting the superiority of the syn conformation in promoting the chain-walking process. Furthermore, Li et al. introduced nitrogen-containing heterocycles as *ortho*-substituents on the N-aryl rings in complexes **7** (**7a** and **7b**) [31]; in these systems, a direct secondary coordination interaction was established between the heterocyclic nitrogen atom and the nickel center. It has been experimentally demonstrated that the thus-constructed “second coordination sphere” effectively modulated the catalytic behavior, drastically suppressing the branching density to less than 1 branch per 1000 carbon atoms and elevating the melting point to above 130 °C, thereby affording nearly linear polymer materials.

#### 3.1.3. Exploration of Nickel Complexes Bearing Bulky Benzhydryl and Derivative Substituents

**Figure 5:**
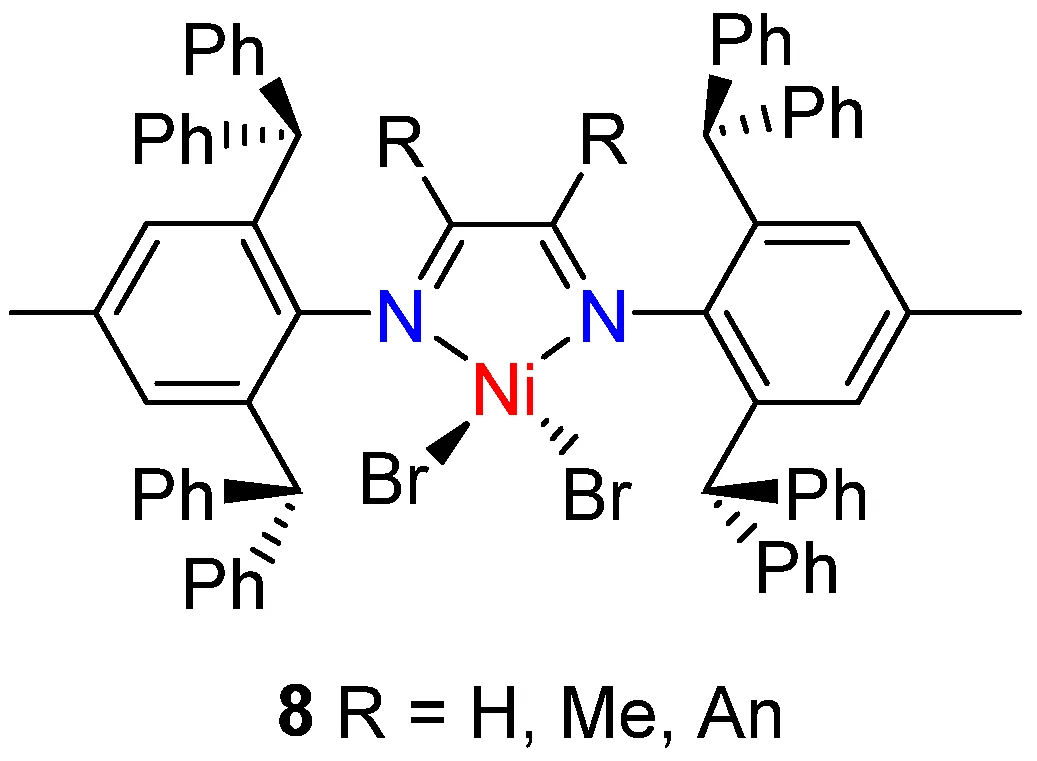
2,6-Diphenylmethyl substituted symmetric *α*-diimine nickel(II) complex **8**.

To further enhance catalyst thermal stability, sterically bulky benzhydryl groups and their derivatives have been extensively introduced into catalytic systems. In 2013, Rhinehart et al. first reported complex **8** (Figure 5), which incorporates *ortho*-benzhydryl substituents on the N-aryl rings and exhibits outstanding performance [32,33,34]. When the backbone substituent R is methyl, this complex displays remarkable thermal stability, retaining a catalytic activity of up to 2.85 × 10^6^ g⋅mol^−1^⋅h^−1^ even at 100 °C (10 min, 100 psi), and yields polyethylene with high molecular weight (*M_n_* up to 6.25 × 10^5^ g⋅mol^−1^), a narrow molecular weight distribution (PDI ≤ 1.46), and a moderate branching density (63–75 branches per 1000 carbon atoms) [32]. Upon further replacement of the backbone with a more rigid acenaphthyl group (R = An), both the thermal stability of the catalyst and the molecular weight of the resulting polymer are significantly improved [34].

**Figure 6:**
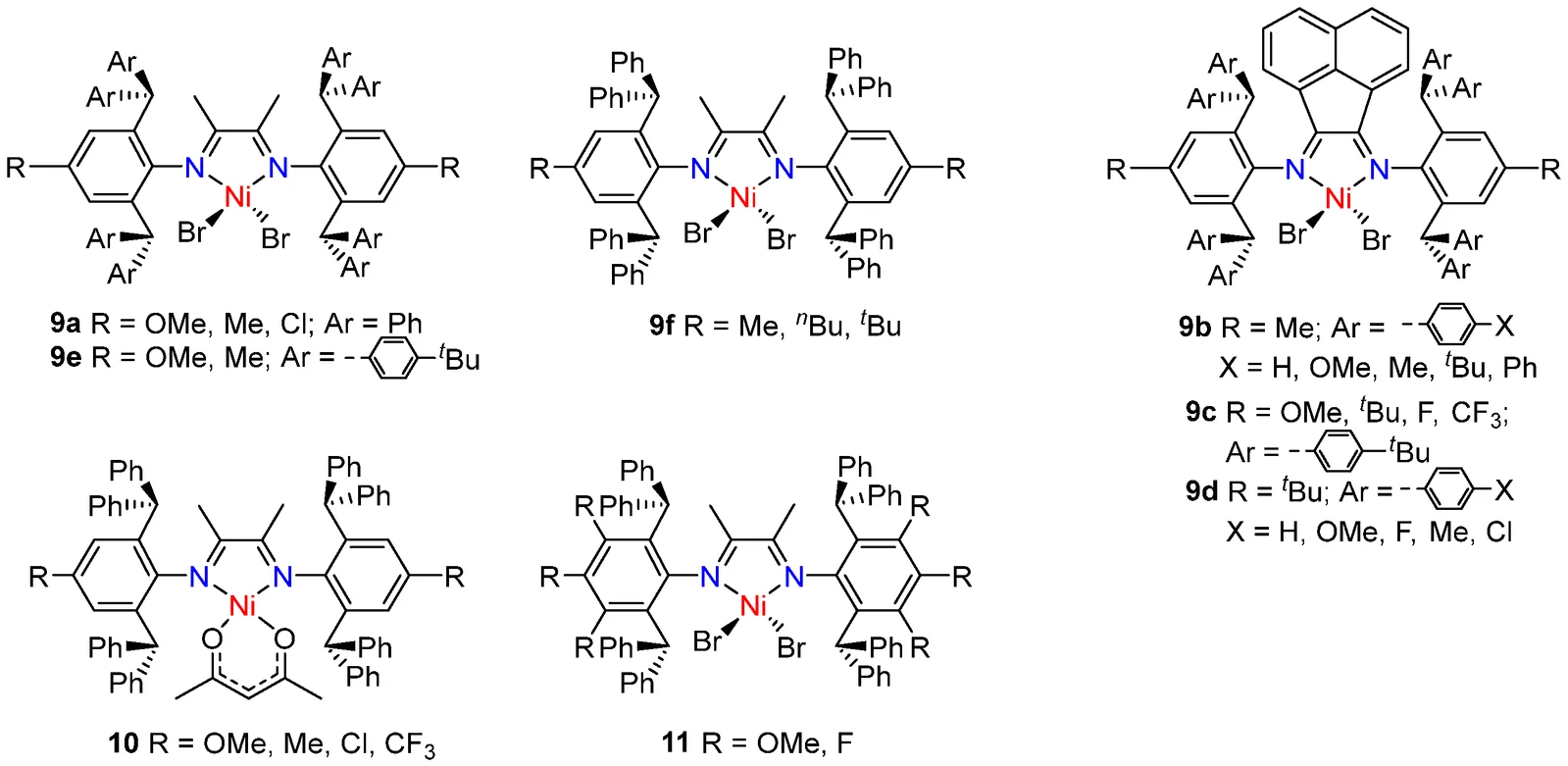
2,6-Diphenylmethyl substituted symmetric *α*-diimine nickel(II) complexes **9**–**11**.

Subsequently, complexes **9**, **10**, and **11** (Figure 6), which feature fully flanked bis-*ortho*-benzhydryl substitution, were developed to further enhance steric protection of the metal center and to systematically investigate the influence of electronic effects imparted by *para*-substituents on the N-aryl rings and by remote substituents on the benzhydryl groups on catalytic performance [35,36,37,38,39,40]. Among them, both complexes **9** and **10** displayed excellent catalytic activity and outstanding thermal stability. For instance, complex **9a** reaches a maximum activity of 6.18 × 10^6^ g⋅mol^−1^⋅h^−1^ (100 °C, 30 min, 0.9 MPa), yielding semicrystalline polyethylene with high molecular weight (*M_n_* up to 1.64 × 10^6^ g⋅mol^−1^) and narrow molecular weight distribution (PDI = 1.1–2.1). Notably, complex **10**, which incorporates trifluoromethyl (CF_3_) groups for electronic tuning, retained extremely high catalytic activity even at elevated polymerization temperatures. On the other hand, Muhammad et al. reported complex **11**, in which multiple methoxy or fluorine substituents were simultaneously introduced at the meta and para positions of the N-aryl rings [40]; only the methoxy-substituted analogue proved catalytically active (5.00 × 10^6^ g⋅mol^−1^⋅h^−1^) (100 °C, 30 min, 0.9 MPa) and capable of producing polymers with ultrahigh molecular weight (*M_n_* up to 25.6 × 10^5^ g⋅mol^−1^), a high melting point (*T_m_* = 100.6 °C), and a low branching density (28–62/1000C). However, the catalyst bearing six fluorine atoms at analogous positions completely lost catalytic activity owing to its facile structural decomposition.

To further expand the boundaries of the mechanical properties of the resulting polymers, Guo et al. reported a family of complexes **9b** bearing remote substituents (H, OMe, Me, ^t^Bu, Ph) at the para positions of the benzhydryl phenyl rings [36], and demonstrated that these complexes exhibit catalytic activities as high as 2.77 × 10^6^ g⋅mol^−1^⋅h^−1^ (100 °C, 30 min, 0.7 MPa) and outstanding thermal stability, affording ultrahigh-molecular-weight polyethylene (*M_w_* up to 45.1 × 10^5^ g⋅mol^−1^) with moderate-to-high branching densities (26–71/1000C). Among them, the complex containing a distal *tert*-butyl substituent yielded polyethylene with a strain recovery (SR) of up to 62%. The introduction of the *tert*-butyl group not only enhanced the mechanical properties of the polymer but also markedly improved the solubility of the catalyst in nonpolar aliphatic hydrocarbon solvents at the molecular level. Building on these findings, Zhu et al. and Li et al. further designed and synthesized complexes **9c** [37] and **9e** [38], each bearing multiple *tert*-butyl groups. Complex **9c** displayed high thermal stability and high catalytic activity, achieving a maximum activity of 7.04 × 10^6^ g⋅mol^−1^⋅h^−1^ (120 °C, 30 min, 3 MPa), and produced ultrahigh-molecular-weight polyethylene elastomers (*M_n_* up to 33.3 × 10^5^ g⋅mol^−1^) with good mechanical properties (SR up to 75%). In contrast, **9e** exhibited slightly lower catalytic activity than **9c** but still possessed good thermal stability and generated polyethylene elastomers with high molecular weight (*M_n_* up to 5.60 × 10^5^ g⋅mol^−1^), high branching density (74–83/1000C), and an SR of up to 84%. Furthermore, complex **9d**, obtained by introducing a *tert*-butyl group at the para position of the N-aryl ring, showed stable high activity over a broad temperature window of 30–100 °C, with catalytic activity consistently remaining on the order of 10^6^ g⋅mol^−1^⋅h^−1^. Based on the above experimental results, the authors propose that this excellent thermal stability is primarily attributed to the π-π stacking interactions between the acenaphthyl backbone and the phenyl rings of the benzhydryl groups, which effectively suppress rotation of the N-aryl moieties, thereby protecting the metal center through axial steric hindrance and enhancing the thermal stability of this series of complexes.

**Figure 7:**
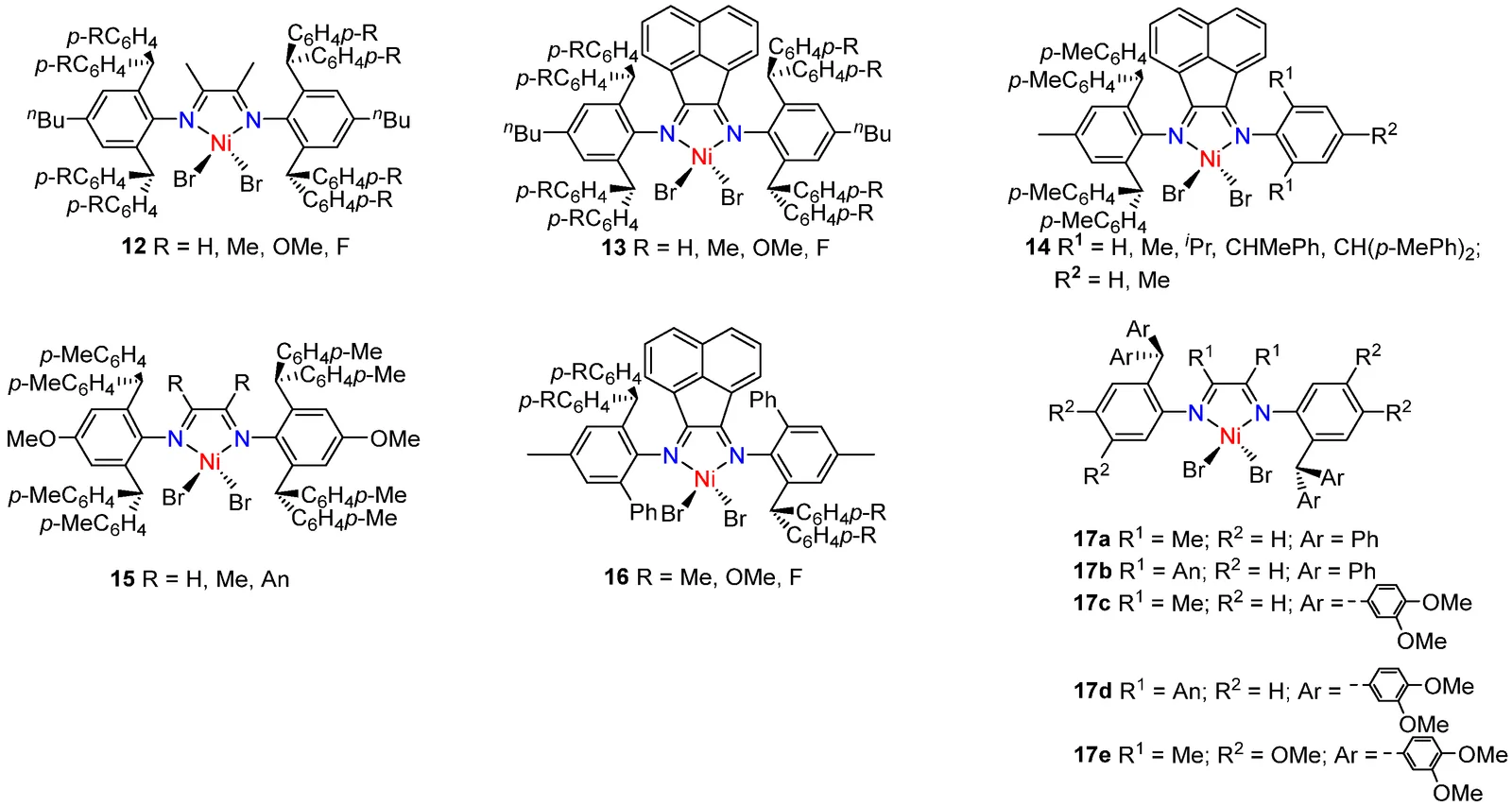
*α*-diimine nickel (II) complexes **12**–**17** containing remote substituent groups.

To further explore the influence of the type and position of remote substituents on the benzhydryl groups of α-diimine nickel(II) complexes on catalytic performance, researchers have successively developed nickel complexes **12**–**17** (Figure 7) modified with diverse remote substituents [41,42,43,44,45,46,47]. Upon introducing a sterically demanding n-butyl group at the para position of the N-aryl ring, the electronic effects of the remote substituents (e.g., hydrogen, methyl, methoxy, and fluorine) begin to exert a remote regulatory influence on the performance of complexes **12** and **13**. Complex **12** generally exhibits high catalytic activity on the order of 10^6^ g⋅mol^−1^⋅h^−1^, with the activity reaching a maximum at 80 °C; the resulting polymer attains a molecular weight (*M_n_*) as high as 8.45 × 10^5^ g⋅mol^−1^, a branching density ranging from 40 to 78 branches per 1000 carbon atoms, and a strain recovery (SR) of up to 80%. Notably, the incorporation of electron-withdrawing remote substituents significantly promotes chain propagation while effectively suppressing chain transfer [41]. The corresponding complex **13**, although displaying catalytic activity comparable to that of **12**, yields polymers with a reduced branching density of 17–53 and a correspondingly increased melting point of 67–127 °C [42].

After breaking the symmetry of the catalyst structure, the unsymmetrical complex **14** bearing a remote methyl group was successfully synthesized [43]. By tuning the substituent type on the other, less sterically encumbered N-aryl ring, the catalytic activity could be modulated over a wide range of (2.8–131.5) × 10^4^ g·mol^−1^·h^−1^, and the resulting polymers exhibited branching densities between 28 and 81 and melting points ranging from −4.7 to 122.9 °C. Among the remote-methyl-substituted complexes **15** featuring different ligand backbones, the acenaphthyl α-diimine nickel(II) complex (R = An) displayed the highest catalytic activity and afforded the highest-molecular-weight polymer, whereas the H-substituted analogue showed low or even no catalytic activity [44]. In complex **16**, which was designed by deliberately attenuating the steric bulk of one N-aryl substituent, the catalyst still maintained outstanding activity on the order of 10^6^ g·mol^−1^·h^−1^ [45]; the resulting polymers attained a molecular weight (*M_n_*) of (0.76–3.52) × 10^4^ g·mol^−1^, a moderate branching density (55–77/1000C), and remarkably superior tensile mechanical properties-elongation at break as high as 704–1097%, tensile strength of 7–25 MPa, and strain recovery (SR) retained at a commendable level of 48–80%. In stark contrast, upon introducing a distal methoxy group into an analogue of complex **16**, the catalytic efficiency declined substantially, and the molecular weight, branching density, and mechanical properties of the resulting polymer all deteriorated comprehensively. Complex **17**, featuring further reduced ortho steric hindrance, upon activation with MAO persistently maintained an exceptional catalytic activity at the level of 10^6–^10^7^ g·mol^−1^·h^−1^ and exhibited distinct temperature-responsive behavior [46]: at low temperatures it tended to generate highly linear polyethylene, whereas at elevated temperatures it switched rapidly to producing highly branched oligomers.

**Figure 8:**
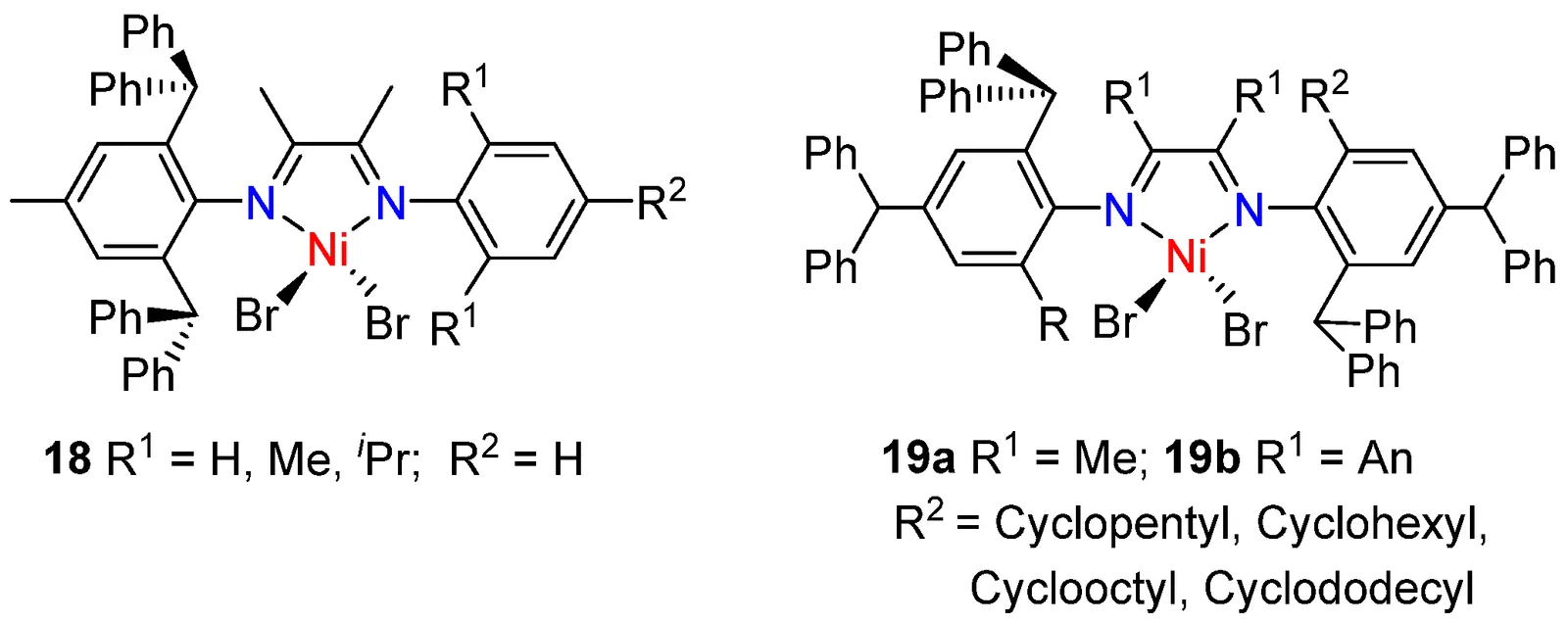
2,6-diphenylmethyl substituted asymmetric *α*-diimine nickel(II) complexes **18**–**19**.

By systematically modulating the number and positions of benzhydryl groups on the N-aryl rings and incorporating various substituents with distinct electronic or steric effects, a series of structurally elaborate catalysts, complexes **18**–**25** (Figure 8), were designed and synthesized. For complex **18**, in which a benzhydryl group is introduced at only one ortho position of the N-aryl ring, studies demonstrated that as the steric hindrance around the active center increases, the branching density of the resulting polyethylene exhibits a decreasing trend [47]. Building on this, researchers introduced a range of cycloalkyl groups of varying ring sizes (from cyclopentyl to cyclododecyl) at the ortho positions of the N-aryl rings to obtain complex **19** [48,49]. Complex **19a** reached the highest activity, 13.2 × 10^6^ g⋅mol^−1^⋅h^−1^ (40 °C, 30 min, 1 MPa), yielding polyethylene elastomers with high molecular weight (*M_w_* = 9.68 × 10^5^ g⋅mol^−1^), narrow molecular weight distribution, and high branching density (73–104/1000C). Notably, the complex **19a** series exhibited exceptionally high selectivity for the formation of short-chain methyl branches, endowing the materials with outstanding elongation at break (353–861%) and favorable strain recovery (SR, 47.4–71.2%). With the introduction of the acenaphthenequinone backbone, the activity of complex **19b** was further enhanced, reaching a maximum of 24.4 × 10^6^ g mol^−1^ h^−1^ (30 °C, 30 min, 1 MPa), albeit with a decrease in the molecular weight of the resulting polyethylene (*M_w_* =6.0 × 10^5^ g⋅mol^−1^) [49].

**Figure 9:**
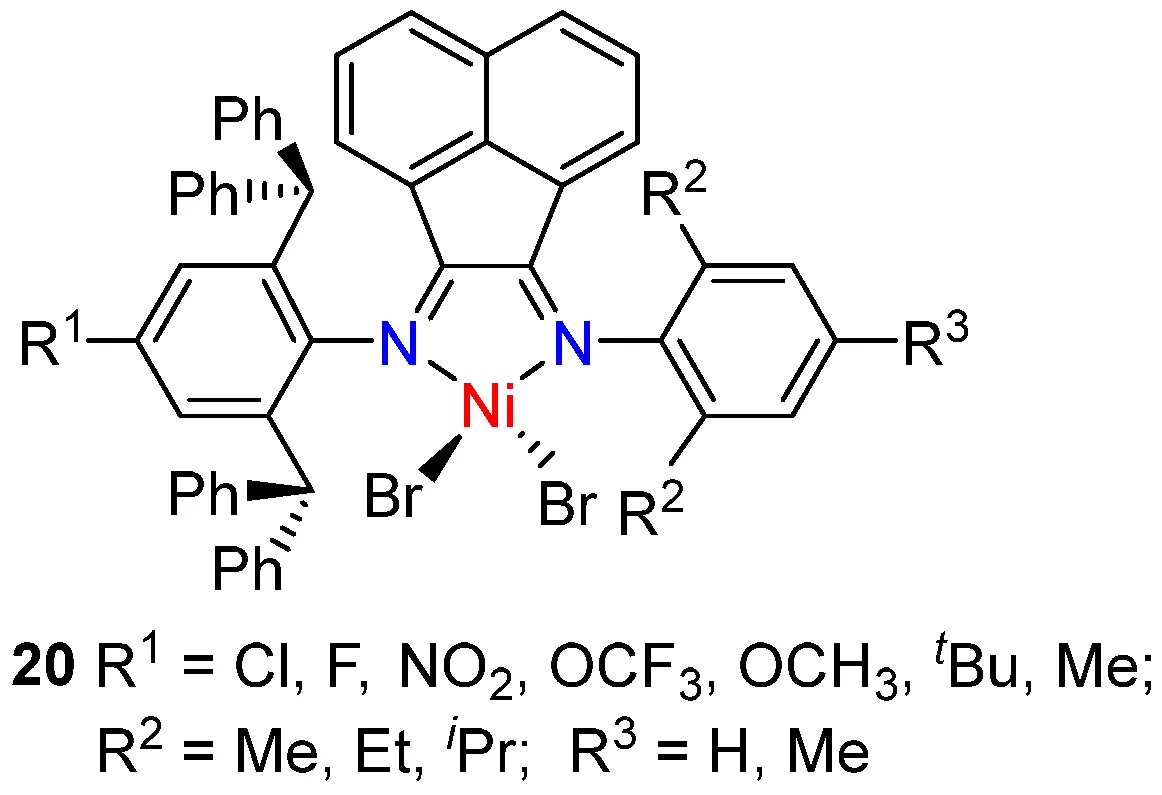
2,6-diphenylmethyl substituted asymmetric *α*-diimine nickel(II) complex **20**.

A series of complexes **20**, incorporating diverse *para*-substituents on the N-aryl rings-including methyl, *tert*-butyl, methoxy, trifluoromethoxy, chloro, fluoro, and nitro groups-were investigated to probe their electronic effects [50,51,52,53,54,55]. These complexes displayed markedly different catalytic activities in toluene. Among them, the methoxy-substituted catalyst exhibited the optimal performance, achieving an activity as high as 17.1 × 10^6^ g⋅mol^−1^⋅h^−1^ (20 °C, 5 min, 1 MPa) [52]. Overall, the presence of electron-donating groups proved more beneficial than electron-withdrawing groups in enhancing the catalytic activity and thermal stability of the complexes, owing to the ability of the electron-donating groups’ positive electronic effect to stabilize the electrophilic metal center. In determining the polymer molecular weight, however, electron-withdrawing groups exhibited a more pronounced advantage: the nitro-substituted complex **20** (Figure 9) yielded the highest molecular weight, and the molecular weight order followed the clear trend nitro > *tert*-butyl > methoxy > fluoro. Notably, the branching density of the products followed an exactly opposite trend, further highlighting the delicate modulation of the chain-walking process by electronic effects. In addition, the catalytic performance of the trifluoromethoxy-substituted complex **20** was examined in different solvents. Its catalytic activity in hexane was found to be substantially higher than that in toluene, reaching a maximum of 21.0 × 10^6^ g⋅mol^−1^⋅h^−1^ (30 °C, 30 min, 1 MPa) [56], and the resulting polymer exhibited a higher branching density (186/1000C) than that obtained in toluene, ultimately affording a polymer with favorable mechanical properties-elongation at break (ε) up to 984% and strain recovery (SR) up to 71%.

**Figure 10:**
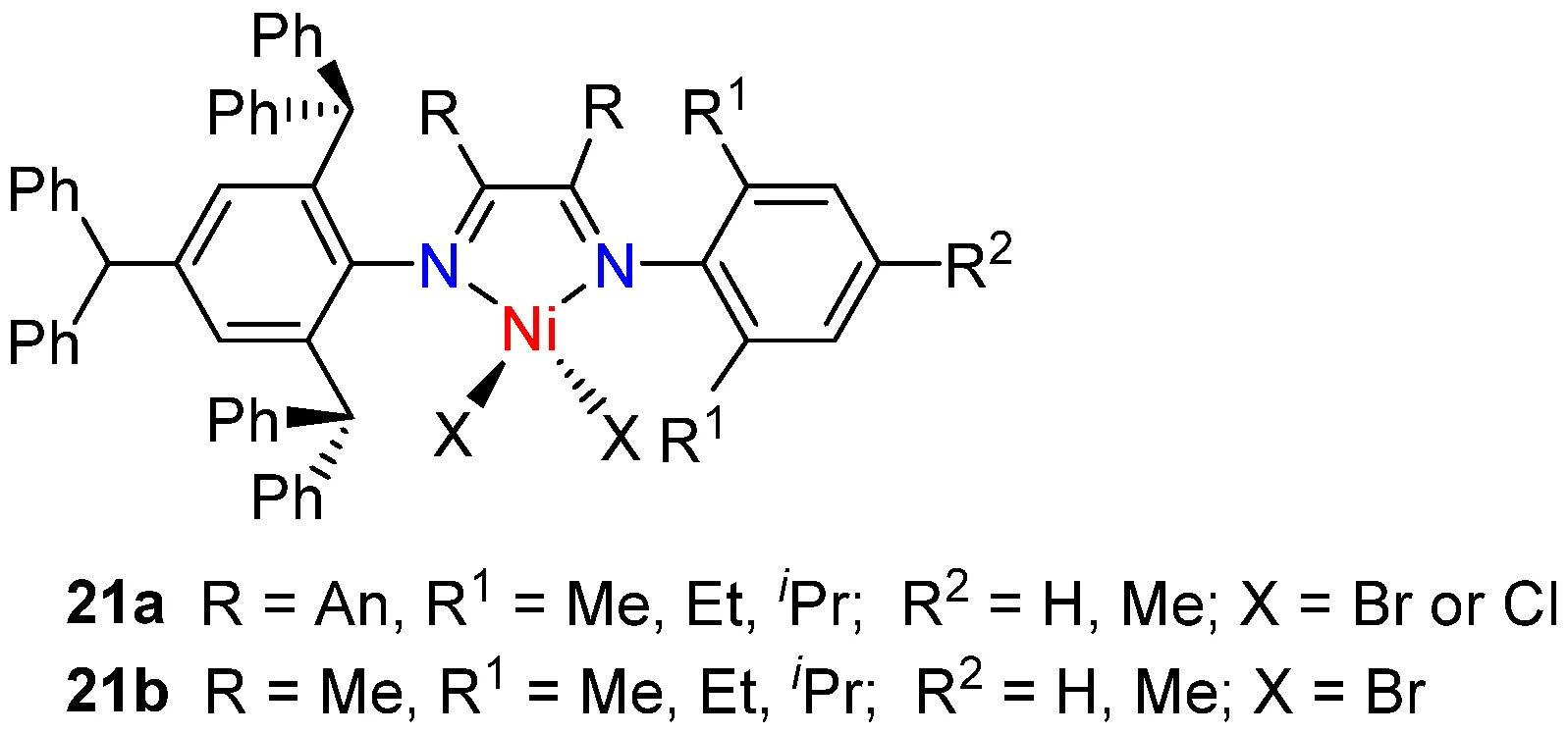
2,6-diphenylmethyl substituted asymmetric *α*-diimine nickel(II) complex **21**.

Upon further increasing the number of benzhydryl substituents on the N-aryl ring, complex **21a** (Figure 10), which bears a 2,4,6-tris(benzhydryl)phenyl moiety, exhibits outstanding catalytic activity (up to 10.7 × 10^6^ g⋅mol^−1^⋅h^−1^; 30 °C, 30 min, 1 MPa), good thermal stability (retaining an activity of 2.97 × 10^6^ g⋅mol^−1^⋅h^−1^ at 90 °C), and yields hyperbranched polyethylene (179/1000C) with a strain recovery of up to 84%, an elongation at break of up to 731% [57]. In contrast, the steric effect exerted by multiple benzhydryl groups is even more pronounced in complex **21b**, which displays high thermal stability over a broad temperature range of 30–100 °C and still achieves a catalytic activity of 1.7 × 10^6^ g⋅mol^−1^⋅h^−1^ at 100 °C [58].

**Figure 11:**
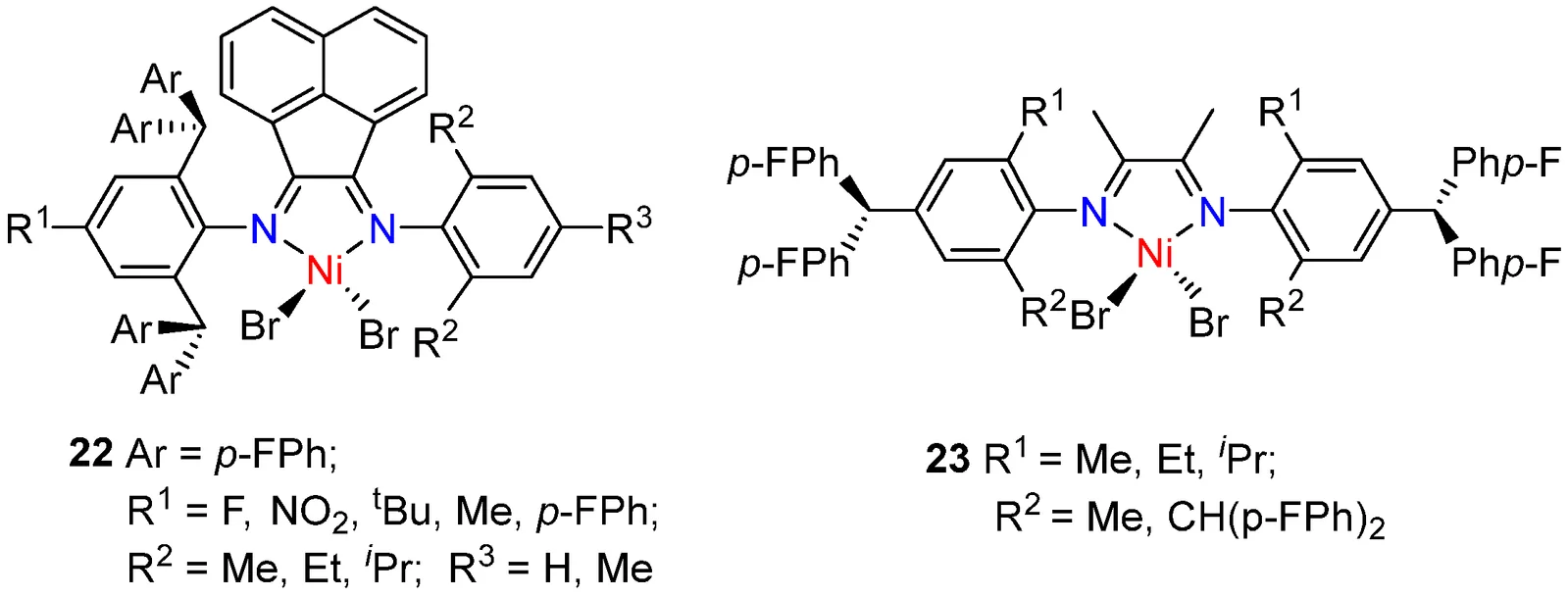
2,6-diphenylmethyl derivatives substituted asymmetric *α*-diimine nickel(II) complexes **22**–**23**.

To further elucidate the role of remote fluorine atoms, the Sun group prepared a series of unsymmetrical complexes **22** (Figure 11) [59,60,61,62,63]. Among these, complex **22** (R^1^ = NO_2_), which contains both nitro and fluorine electron-withdrawing groups, exhibits far higher catalytic activity (6.01 × 10^6^ g⋅mol^−1^⋅h^−1^; 80 °C, 30 min, 1 MPa) and thermal stability than the analogous complex **22** (R^1^ = NO_2_) that lacks the bis(4-fluorophenyl)methyl substituent [54]. Furthermore, the highly active complex **23**, which incorporates the bis(4-fluorophenyl)methyl group, achieves a catalytic activity up to 10.0 × 10^6^ g⋅mol^−1^⋅h^−1^ and tends to afford high-performance polymers richly populated with short-chain branches (methyl branching fraction exceeding 87%), a molecular weight (*M_w_*) of 15.19 × 10^5^ g⋅mol^−1^, and a branching density in the range of 86–102 branches per 1000 carbon atoms [64]. Specifically, the presence of abundant short-chain branches (e.g., methyl, ethyl) disrupts crystallinity, while the residual crystallites can still function as physical crosslinking points. A higher proportion of long-chain branching promotes chain entanglements and enhances strain-induced crystallization, thereby imparting excellent elastic recovery. Furthermore, the presence of long-chain branches significantly enhances shear thinning and melt strength, which are critical for film blowing and foam extrusion processes.

**Figure 12:**
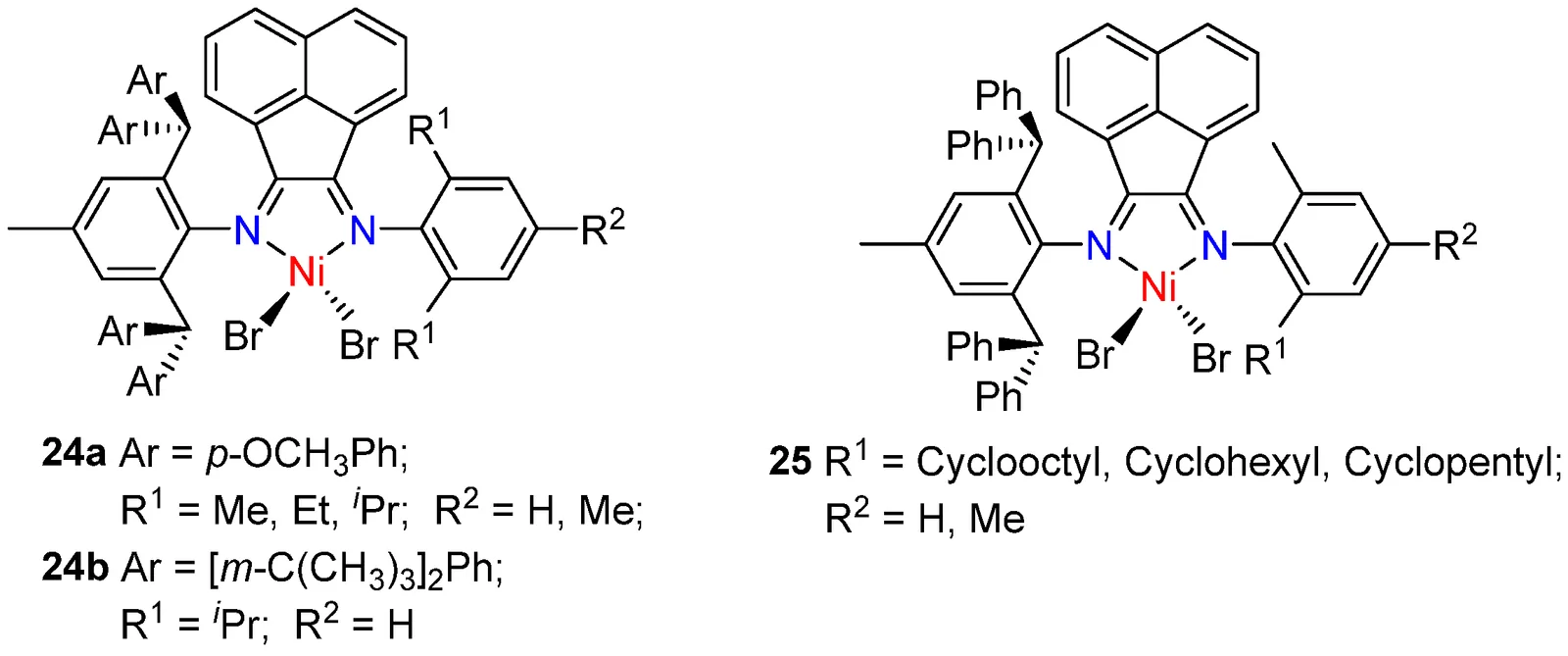
2,6-diphenylmethyl derivatives substituted asymmetric *α*-diimine nickel(II) complexes **24**–**25**.

Electron-donating groups likewise impart outstanding performance when deployed as remote substituents. Complex **24a** (Figure 12), which bears a remote methoxy group, is highly sensitive to the choice of cocatalyst: with Et_2_AlCl, the branching density of the product sharply increases to 115 branches per 1000 carbon atoms, the elongation at break reaches 553.5%, and the strain recovery (SR) attains a value as high as 84%; switching to MAO causes the branching density to drop abruptly to 29 [65]. Upon introduction of a meta-*tert*-butyl group, complex **24b** experiences a concurrent increase in both steric hindrance and electron density, which markedly improves its solubility in aliphatic hydrocarbons and, in turn, significantly enhances the thermal stability and the molecular weight of the resulting polymer [66]. In the complex **25** system, which combines benzhydryl groups with various cycloalkyl substituents, the catalytic activity reaches up to 17.5 × 10^6^ g⋅mol^−1^⋅h^−1^ (30 °C, 30 min, 1 MPa), and the molecular weight (*M_n_*) of the resulting branched polyethylene elastomers can be regulated within the range of (2.14–6.68) × 10^5^ g⋅mol^−1^. Among these, the bulkiest cyclooctyl group imparts the highest catalytic activity, further confirming the principle that tighter axial shielding of the metal center accelerates the chain propagation rate [67].

**Figure 13:**
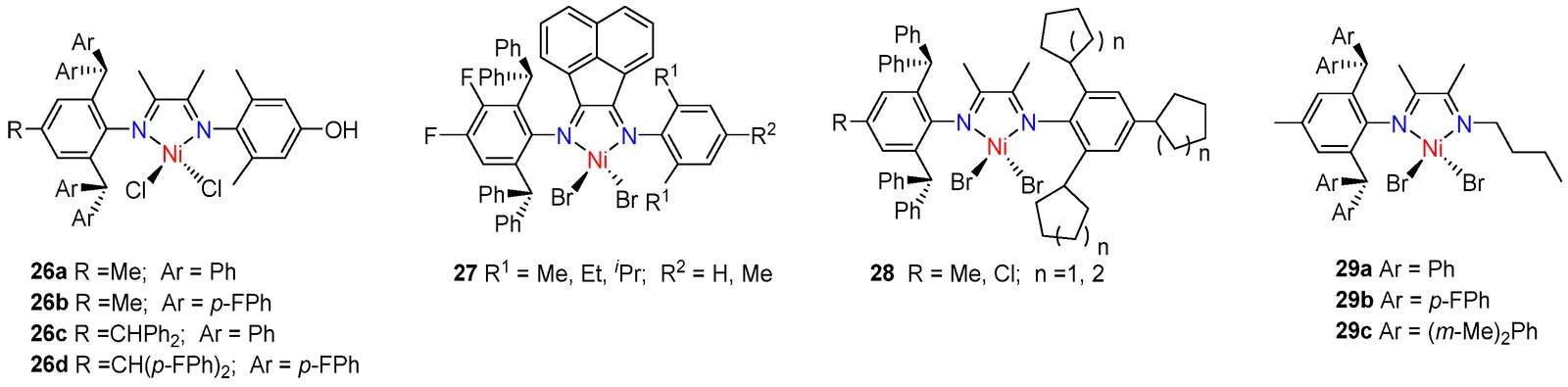
Stereoscopically or electronically modified asymmetric *α*-diimine nickel(II) complexes **26**–**29**.

Complex **26** (Figure 13), featuring an unsymmetrical architecture with a *para*-hydroxy group on the N-aryl ring and a highly imbalanced steric environment, effectively modulates the microscopic competition between monomer insertion and metal chain walking, ultimately delivering a remarkable catalytic activity of 29.1 × 10^6^ g⋅mol^−1^⋅h^−1^ and yielding an ultrahigh-molecular-weight polymer with a molecular weight (*M_w_*) of 1.81 × 10^6^ g⋅mol^−1^ [68]. Notably, the terminal free hydroxyl group, far from poisoning the metal center, provides a convenient handle for subsequent covalent grafting of the catalyst onto solid support surfaces. Complex **27**, which bears a difluoro modification, also exhibits good catalytic activity (5.90 × 10^6^ g⋅mol^−1^⋅h^−1^; 30 °C, 30 min, 1 MPa), and the resulting elastomer achieves an elongation at break as high as 2592% under a tensile stress of 0.97 MPa [69]. The presence of flexible cycloalkyl groups in complex **28** facilitates chain propagation, endowing it with high catalytic activity (13.6 × 10^6^ g⋅mol^−1^⋅h^−1^; 50 °C, 10 min, 0.6 MPa) and the capability to produce polyethylene elastomers with high molecular weight (*M_n_* up to 9.67 × 10^5^ g⋅mol^−1^), high branching density (70–92/1000C), and a strain recovery (SR) of up to 83% [70]. Complex **29**, in which one rigid N-aryl ring is replaced by a highly flexible alkyl chain, significantly reduces the steric hindrance on the opposite side of the active center. As a result, it exhibits high catalytic activity (substantially exceeding 10^6^ g⋅mol^−1^⋅h^−1^) and generates low-molecular-weight polyethylene (*M_n_* = 1–10 kg·mol^−1^) with high branching density (66–95/1000C) [71].

**Figure 14:**
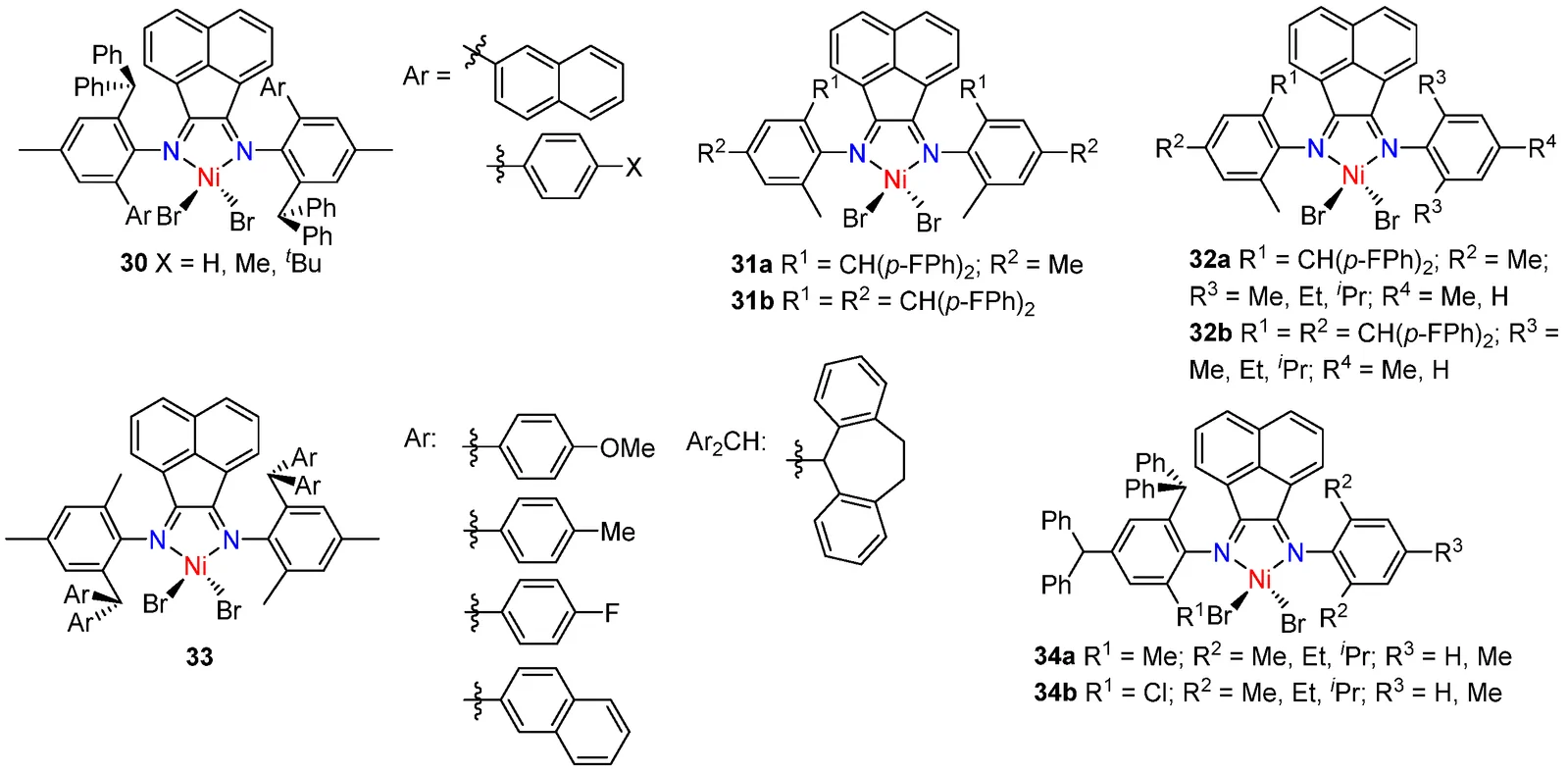
Single *ortho*-diphenylmethyl and its derivatives substituted acenaphthyl *α*-diimine nickel(II) complexes **30**–**34**.

Researchers applied the same steric-hindrance-reducing strategy to the acenaphthene backbone system and, by substantially lowering the ortho steric bulk on one N-aryl side while retaining only a single benzhydryl group, designed and synthesized complexes **30**–**34** (Figure 14). Among these, complex **30** exhibited a high activity of 8.80 × 10^6^ g⋅mol^−1^⋅h^−1^ (30 °C, 30 min, 0.8 MPa); although the molecular weight (*M_n_*) of the resulting polyethylene dropped to 5.78 × 10^4^ g⋅mol^−1^, its branching density increased considerably [72]. Complexes **31** and **32**, which bear a single *ortho*-bis(4-fluorophenyl)methyl group, both displayed activities exceeding 1 × 10^6^ g⋅mol^−1^⋅h^−1^ when activated with MAO or EASC; notably, complex **32b** retained an activity of 1.12 × 10^6^ g⋅mol^−1^⋅h^−1^even under the demanding condition of 100 °C [73,74]. Complex **33**, which carries a single *ortho*-benzhydryl derivative, effectively suppressed chain transfer owing to the “restricted rotation” effect, achieving a high activity of 5.32 × 10^6^ g⋅mol^−1^⋅h^−1^ at 80 °C and affording polyethylene that combines high molecular weight with a tunable branching density (15–77/1000C) and a strain recovery (SR) of up to 76% [75]. Complex **34a**, bearing an *ortho*-methyl substituent, reached a maximum activity of 8.95 × 10^6^ g⋅mol^−1^⋅h^−1^ at 20 °C (20 °C, 30 min, 1 MPa) and, at 80 °C, produced a polymer with a branching density as high as 166 [76]; its *ortho*-chloro substituted analogue **34b** exhibited even higher catalytic activity and thermal stability, attaining 10.9 × 10^6^ g⋅mol^−1^⋅h^−1^ (50 °C, 30 min, 1 MPa) [76].

**Figure 15:**
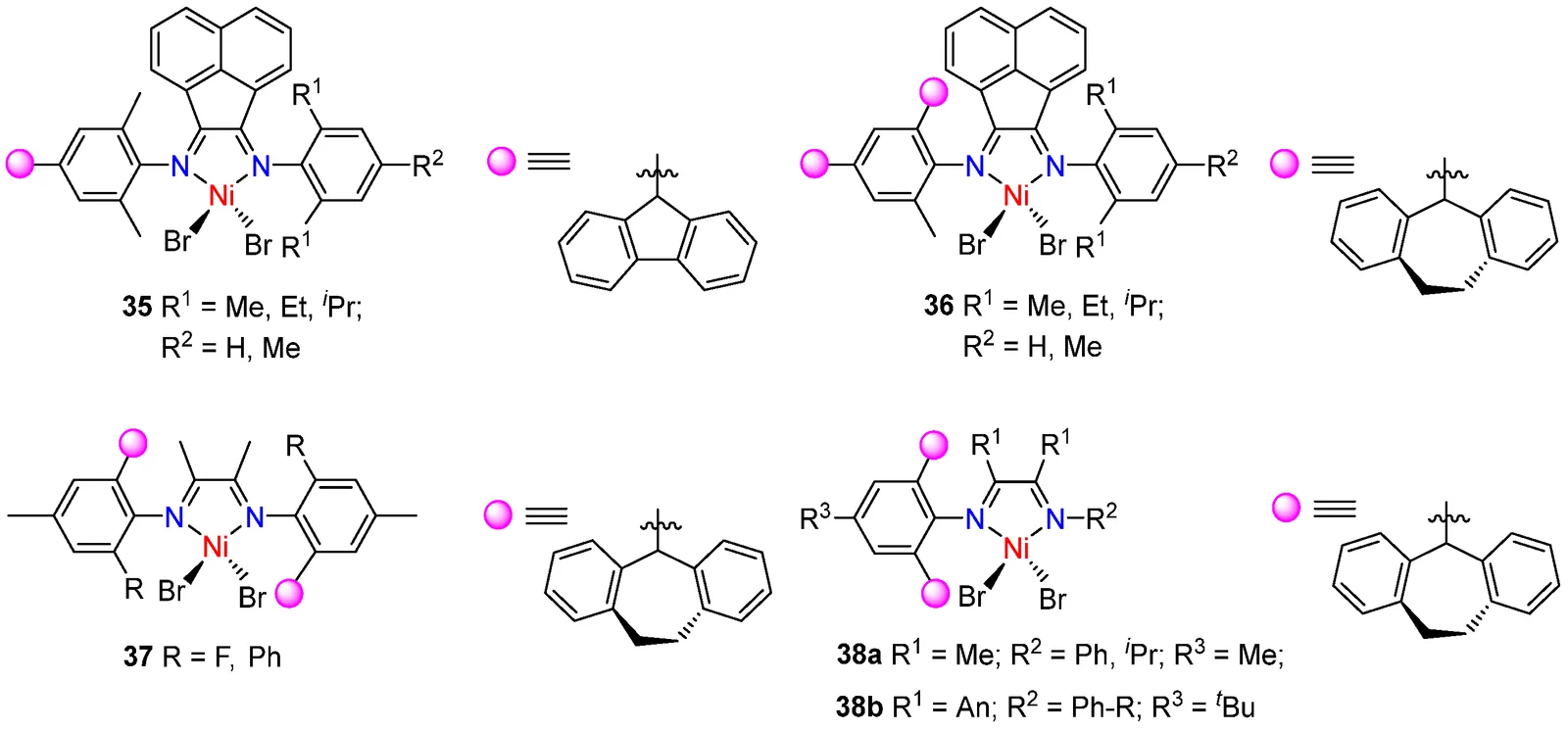
Acenaphthyl *α*-diimine nickel(II) complexes **35**–**38** containing dibenzocyclopentyl and dibenzocycloheptyl groups.

To achieve higher polymer molecular weights, researchers introduced bulky rigid groups-such as dibenzocyclopentyl and dibenzocycloheptyl moieties-into the catalyst backbone, leading to the design and synthesis of complexes **35**–**38** (Figure 15). Complex **35**, which carries only one rigid group on a single side, showed a decline in catalytic activity as the steric hindrance on the opposite side increased; however, the molecular weight of the resulting polymer was substantially enhanced [77]. Complex **36**, bearing dibenzocycloheptyl groups on both sides, exhibited high catalytic activity for ethylene polymerization, reaching up to 10.56 × 10^6^ g⋅mol^−1^⋅h^−1^ (30 °C, 30 min, 1 Mpa, Table 1) and affording a polymer with a weight-average molecular weight (*M_w_*) as high as 5.9 × 10^5^ g⋅mol^−1^ and melting points consistently above 95 °C [78]. In complex **37**, where rigid groups are mounted along both axial directions to create a “sandwich” geometry, the rotational freedom is almost entirely suppressed; this effectively inhibits chain transfer and brings about a dramatic increase in polymer molecular weight, albeit at the cost of a sharply reduced catalytic activity [79]. Complex **38a** achieves a favorable balance between activity and molecular weight: it maintains catalytic activity at the 10^6^ g⋅mol^−1^⋅h^−1^ level and yields high-performance polyethylene with a molecular weight (*M_n_*) up to 5.86 × 10^5^ g⋅mol^−1^ and a branching density in the range of 80–93 branches per 1000 carbon atoms [80]. By further increasing the rigidity of the ligand backbone, the thermal stability of complex **38b** was significantly enhanced, allowing it to retain excellent polymerization activity even at a polymerization temperature of 100 °C. The molecular weight of the resulting polyethylene decreased slightly as the polymerization temperature increased [81].

**Figure 16:**
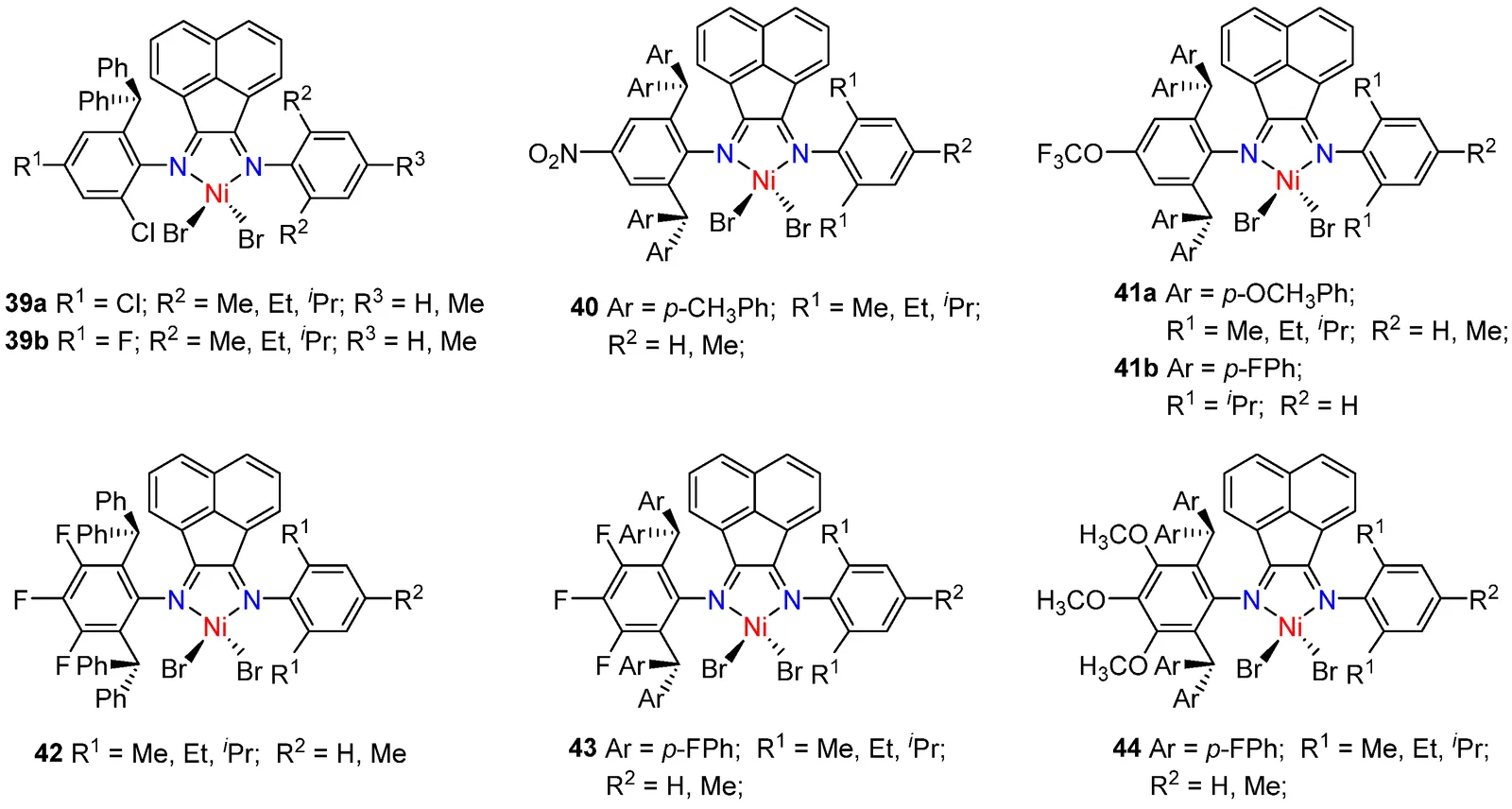
Multiple electronic effects modified asymmetric *α*-diimine nickel(II) complexes **39**–**44**.

Over the past two years, to further fine-tune the catalytic performance of α-diimine nickel catalysts through electronic effects, researchers have introduced various substituents at different positions of the imine aryl rings, designing and synthesizing complexes **39**–**44**. Complex **39** (Figure 16), which bears benzhydryl and chlorine substituents simultaneously at the ortho positions of the N-aryl ring while varying the para substituent as chlorine (**39a**) or fluorine (**39b**), displayed good thermal stability during ethylene polymerization. Among them, **39a** maintained a remarkable activity up to 12.2 × 10^6^ g·mol^−1^·h^−1^ at a polymerization temperature of 50 °C (Table 2) [82]. Although the fluorine-containing **39b** showed a slightly decreased activity, the resulting polyethylene exhibited improvements in molecular weight (*M_w_* = 1.52 × 10^5^ g·mol^−1^), melting point (*T_m_* = 114.7 °C), branching density (62/1000C), and elastic recovery (SR = 55%), indicating that the introduction of fluorine substituents is beneficial for enhancing the overall performance of the elastomeric material [83]. Complex **40**, featuring bulky substituents on both ortho positions together with a strong electron-withdrawing nitro group at the para position, achieved a maximum activity of 11.7 × 10^6^ g·mol^−1^·h^−1^ (30 °C, 30 min, 1 MPa) and produced highly branched polyethylene (121/1000C) [84]. The incorporation of trifluoromethoxy substituents in complex **41** significantly enhanced both catalytic activity and the molecular weight of the resulting polyethylene. For instance, **41b** bearing a remote fluoro substituent displayed an activity up to 21.4 × 10^6^ g·mol^−1^·h^−1^ (40 °C, 30 min, 1 MPa), affording moderately branched (90/1000C) polyethylene with a high molecular weight (*M_w_* = 6.41 × 10^5^ g·mol^−1^) [85]. The introduction of a remote methoxy group in **41a** maintained the high activity and further improved the molecular weight (*M_w_* = 6.41 × 10^5^ g·mol^−1^) and elastic recovery (SR = 68%) [86]. However, the molecular weights of these elastomers are markedly higher than those of commercial POEs, which also gives rise to processing difficulties. Complex **42**, with multiple fluorine substituents introduced at both the meta and para positions of the N-aryl ring, enhanced the chain-walking ability of the catalyst and yielded polyethylene elastomers with high branching (134/1000C) and an outstanding strain-at-break (ɛ = 1797%) [87]. Further incorporating a remote fluoro substituent led to complex **43**, which maintained high activity (up to 10.3 × 10^6^ g·mol^−1^·h^−1^) while producing polyethylene with moderate branching (87/1000C) and good elastic recovery (SR = 52%) [88]. Complex **44**, bearing electron-donating methoxy groups at the meta and para positions, increased the molecular weight of the resulting polyethylene (*M_w_* = 3.20 × 10^5^ g·mol^−1^), and the obtained elastomer retained a good strain-at-break (ɛ = 1251%) [89].

#### 3.1.4. Exploration of Fused-Ring Systems and Sandwich Effects on Nickel Complexes

**Figure 17:**
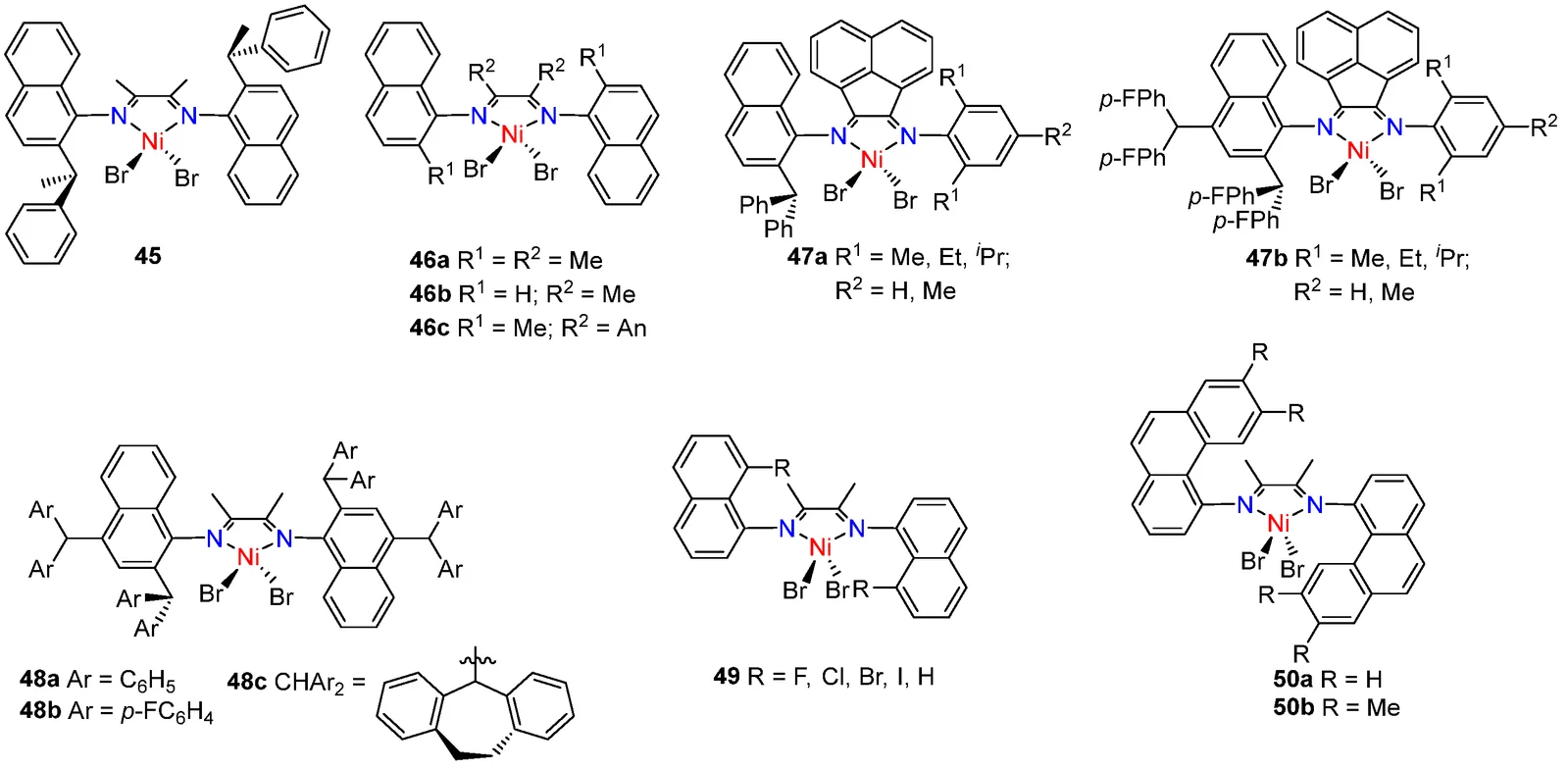
Symmetric and asymmetric *α*-diimine nickel(II) complexes **45**–**50** containing naphthyl group.

Extending their vision from simple benzene rings to the more sterically demanding naphthyl and phenanthryl frameworks (complexes **45**–**60**, Figure 17), researchers sought new breakthroughs by enlarging the aromatic conjugated system. Two families of N-naphthyl complexes, **45** and **46**, which bear different substituents at the ortho positions, both exhibited catalytic activities as high as 2.81 × 10^6^ g⋅mol^−1^⋅h^−1^ (40 °C, 10 min, 0.12 MPa) and produced polyethylene with high branching density (86–117/1000C) [90]. Complex **47a**, featuring an *ortho*-benzhydryl substituent, maintained activity on the order of 10^6^ g⋅mol^−1^⋅h^−1^ while the branching density dropped to 12–61/1000C, and the polymer melting point exceeded 100 °C [91]. In contrast, complex **47b**, which carries two bis(4-fluorophenyl)methyl groups, showed unremarkable activity yet produced highly uniform polymeric material with an extremely narrow molecular weight distribution (PDI = 1.22–1.99) and a molecular weight (*M_n_*) as high as 7.12 × 10^5^ g⋅mol^−1^ [92]. Complex **48**, which is elaborately decorated with benzhydryl groups and their derivatives, delivered outstanding catalytic activity (13.0 × 10^6^ g⋅mol^−1^⋅h^−1^; 30 °C, 10 min, 0.6 MPa) and, relative to complex **45**, yielded polyethylene with higher molecular weight (*M_n_* = (4.46–8.54) × 10^5^ g⋅mol^−1^) and lower branching density (16–40/1000C) [93]. Complexes **49** (containing 8-halonaphthyl groups) and **50** (bearing phenanthryl groups) display catalytic performance that is largely dictated by the interconversion of syn/anti diastereomers [94,95]. Among them, complex **50b**, which exists exclusively as the anti isomer, exhibits a single kinetic regime in polymerization, affording monomodal high-molecular-weight linear polyethylene with a turnover frequency (TOF) of 5.16 × 10^6^ h^−1^ (40 °C, 15 min, 2.72 MPa).

**Figure 18:**
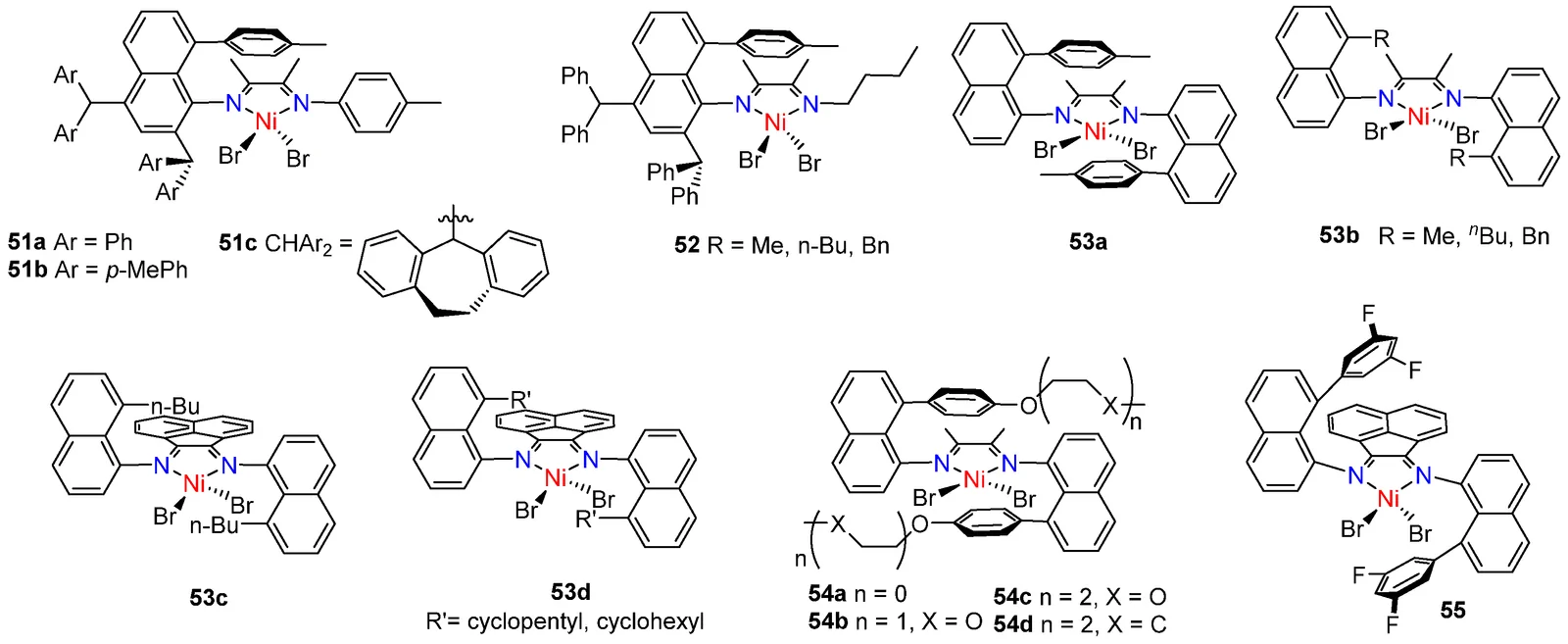
“Half Sandwich” and “Sandwich” type *α*-diimine nickel(II) complexes **51**–**55** containing naphthyl groups.

The so-called “sandwich” or “half-sandwich” type steric modification strategy (complexes **51**–**55**, Figure 18) essentially operates by imposing significant steric hindrance above and below the axial positions to shield the metal center. Complex **51**, which possesses only a single naphthyl substituent, exhibits a moderate activity on the order of 10^5^–10^6^ g⋅mol^−1^⋅h^−1^ yet is capable of yielding soft elastomers with a molecular weight (*M_n_*) as high as 473.2 kg·mol^−1^ and an ultrahigh branching density (146–168/1000C) [96]. Complex **52**, which bears a flexible N-alkyl chain, displays an activity in the 10^5^ range, and both its branching density (74–81/1000C) and molecular weight (*M_n_* up to 207.9 kg·mol^−1^) are slightly lower [70]. Sandwich-type complex **53**, bearing tolyl, alkyl, and cycloalkyl groups at the 8-positions of the two N-naphthyl rings, shows high catalytic activity (3.37 × 10^6^ g⋅mol^−1^⋅h^−1^; 30 °C, 30 min, 0.6 MPa) and produces branched polyethylene (branching density 41–121/1000C) with a broad molecular weight distribution (PDI = 4.6–29.3) [97,98,99]. The “poly(ethylene glycol) sandwich” complex **54** represents a highly creative design: as the PEG segment is lengthened, the activities of complexes **54b**, **54c**, and **54d** increase roughly 9-fold relative to the parent **54a**, and the product can be modulated from a highly branched amorphous state (106/1000C) to a lightly branched semicrystalline state (19/1000C) [100]. A family of sandwich-type complexes **55**, containing electron-withdrawing fluorine groups, displays a unique ability to precisely control the microstructure, opening a new avenue for the synthesis of multi-block copolymers that integrate rigid linear segments with soft branched segments [101].

**Figure 19:**
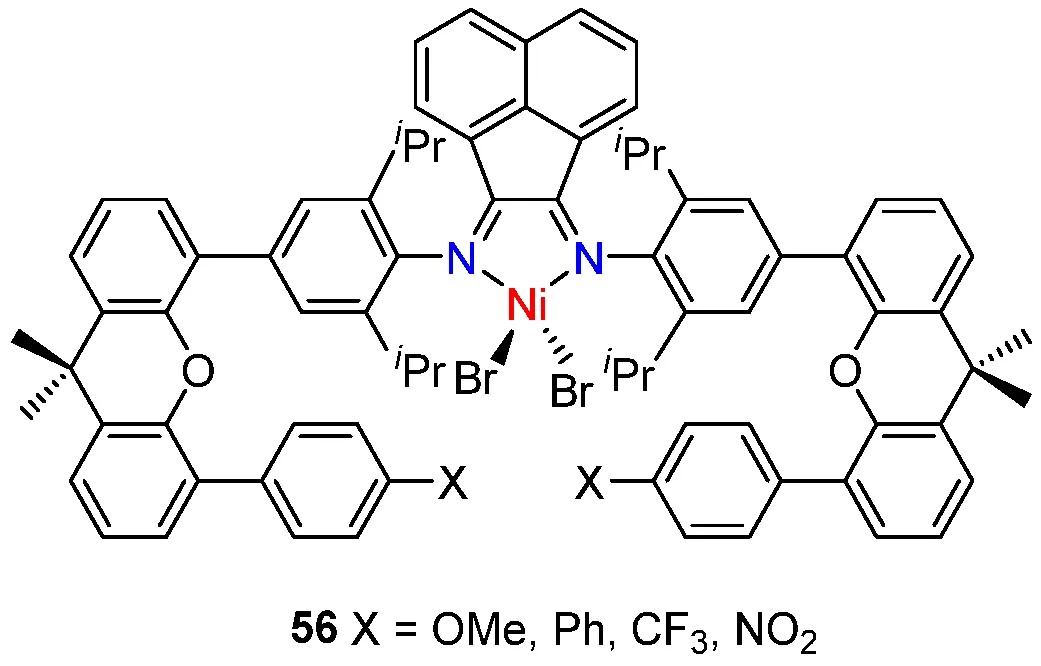
Xanthene-bridged acenaphthyl *α*-diimine nickel (II) complex **56**.

To achieve a more comprehensive, three-dimensional steric shielding, researchers designed and synthesized complex **56** (Figure 19), which incorporates a bulky xanthene group [102]. This complex retains a high catalytic activity of 3.51 × 10^6^ g⋅mol^−1^⋅h^−1^ at 80 °C and yields polymers with a molecular weight (*M_n_*) up to 1.53 × 10^6^ g⋅mol^−1^ and a branching density in the range of 28–101/1000C. Notably, when the substituent X is a phenyl group, the elastic properties of the resulting material are already fully comparable to those of commercially established thermoplastic elastomer products.

#### 3.1.5. Exploration of Multidirectional Steric Modification of Nickel Complex Structures

**Figure 20:**
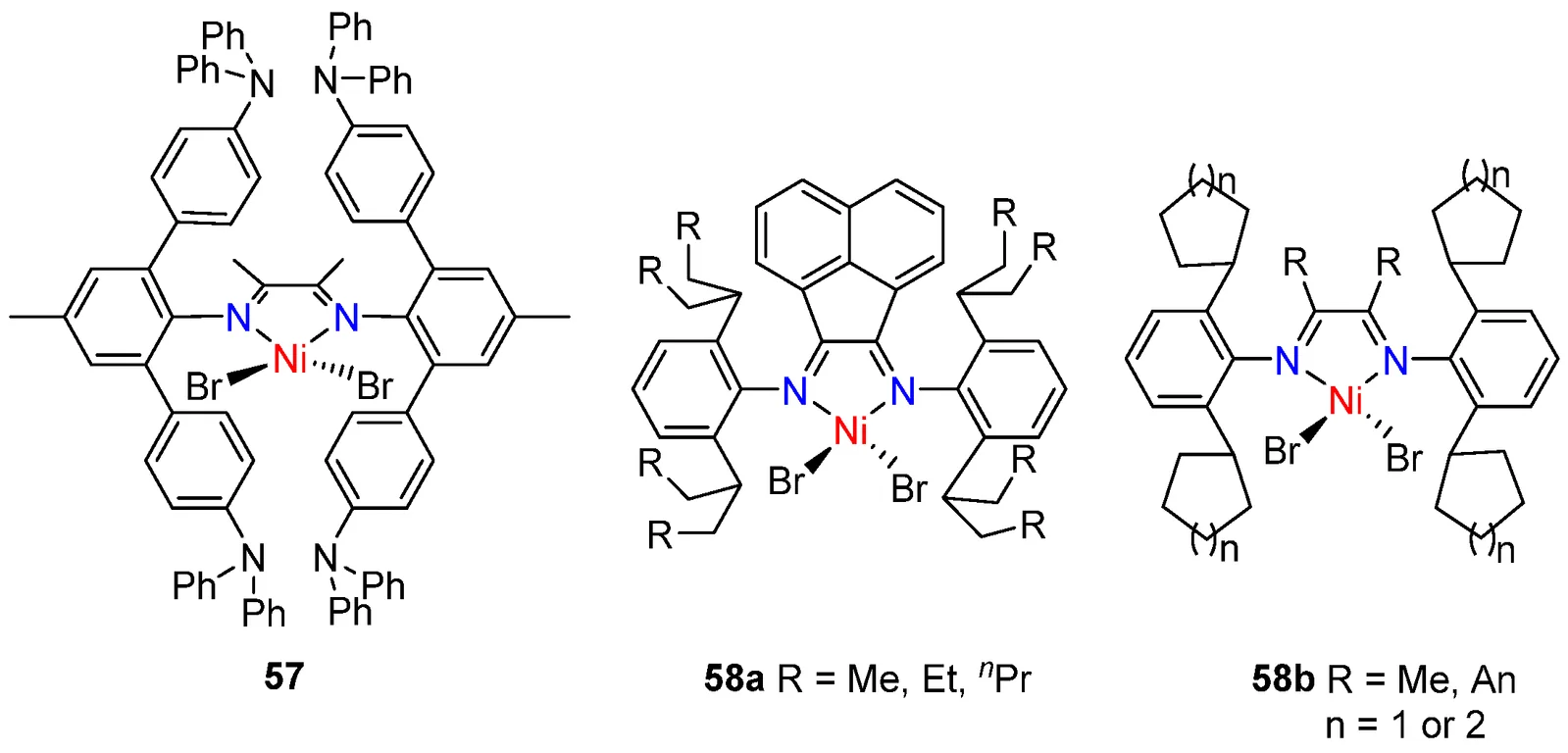
Stereoscopic modified symmetrical *α*-diimine nickel(II) complexes **57**–**58**.

Based on the concept of a synergistic double-layer steric strategy, Xia et al. designed and synthesized complex **57** (Figure 20) [103]. In this complex, the bulky diphenylamine group not only forms a first steric shielding in the inner sphere adjacent to the metal center, but also constructs a peripheral steric barrier that effectively blocks chain transfer reactions. This catalytic system maintains an activity of 2.00 × 10^6^ g·mol^−1^·h^−1^ even at a high temperature of 150 °C, and at 30 °C it exhibits an ultra-high activity of up to 1.03 × 10^9^ g·mol^−1^·h^−1^ (30 °C, 1 min, 3.2 MPa); the resulting polyethylene reaches a molecular weight (*M_w_*) of up to 4.67 × 10^6^ g·mol^−1^, and its branching degree can be finely tuned over the range of 2–32/1000C. Complex **58a**, which bears flexible long-chain alkyl groups, can adopt different conformations and generate dynamic steric hindrance around the metal center, thereby increasing the ethylene insertion barrier in certain cases and consequently lowering the catalytic activity, with the highest activity reaching only 2.14 × 10^6^ g·mol^−1^·h^−1^ (20 °C, 30 min, 0.6 MPa) [104]. This system ultimately produces highly branched polyethylene (branching degree up to 112/1000C) possessing good elasticity (strain recovery, SR, up to 88%). In contrast, complex **58b** containing flexible cycloalkyl groups exhibits slightly improved activity, attaining 4.89 × 10^6^ g·mol^−1^·h^−1^ (50 °C, 30 min, 0.6 MPa), and the resulting polymer similarly displays a high branching density (112/1000C) and a high molecular weight (*M_n_* = 5.44 × 10^5^ g·mol^−1^) [105].

**Figure 21:**
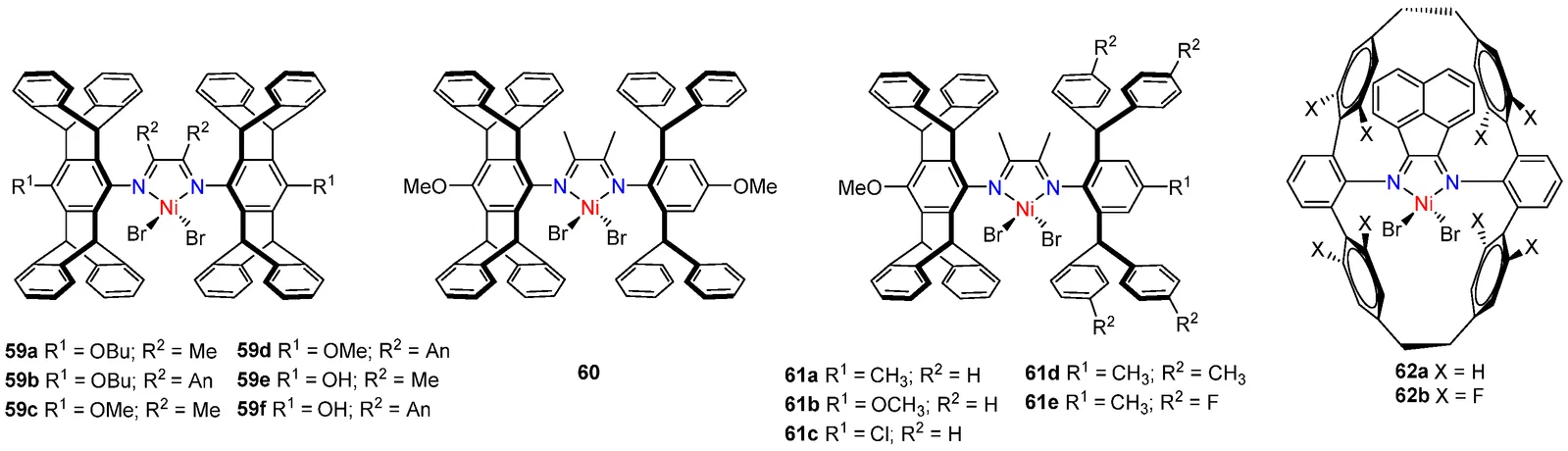
Stereoscopic modified symmetrical *α*-diimine nickel(II) complexes **59**–**62**.

Complexes **59a** and **59b** (Figure 21), which bear extremely bulky bowl-shaped bis(pentenyl) ligands, confine the metal center within an extremely congested cavity constructed by two hemispherical ligands. Both catalysts exhibit outstanding catalytic activities: the former achieves a maximum activity of 34.0 × 10^6^ g·mol^−1^·h^−1^ (60 °C, 30 min, 0.7 MPa). The polyethylenes obtained from both complexes possess low branching densities (<40/1000C), and the maximum number-average molecular weight (*M_n_*) reaches 1.49 × 10^5^ g·mol^−1^; the melting points are both around 130 °C [106]. Complexes **59c**–**59f** with similar structures exhibit activities in the range of 0.64–3.74 × 10^6^ g·mol^−1^·h^−1^, afford polymers with *M_n_* up to 1.05 × 10^6^ g·mol^−1^, and give branching densities between 6 and 56/1000C [107]. The unsymmetrical complex **60**, which combines a pentenyl group and a benzhydryl group, shows an activity of up to 26.5 × 10^6^ g·mol^−1^·h^−1^ (50 °C, 5 min, 0.8 MPa) and yields ultra-high-molecular-weight polyethylene (*M_w_* > 10^6^ g·mol^−1^) [17]. When fine-tuning the electronic effects in the analogous complex **61**, it was found that introducing chlorine substituents slightly increases the activity to 29.2 × 10^6^ g·mol^−1^·h^−1^ (50 °C, 5 min, 0.8 MPa) and tends to produce polyethylene with a high branching density (128/1000C); introducing methyl groups favors the formation of polymer with the highest molecular weight (*M_w_* = 2.57 × 10^6^ g·mol^−1^); whereas the presence of fluorine atoms, although it raises the branching density, lowers both the activity and the molecular weight to different extents [108]. To fundamentally overcome the thermal decomposition problem, complexes **61a** and **61b**, which possess rigid cyclophane backbones that completely restrict the free rotation of the C-N bonds, were developed [109,110]. The fluorine-free **62a** exhibits excellent high-temperature catalytic performance at 90 °C (TOF up to 1.07 × 10^6^ h^−1^) and yields a polymer with *M_n_* = 4.62 × 10^5^ g·mol^−1^; the fluorine-substituted analogue **62b** not only shows further enhanced thermal stability but also catalytically generates a specialty polymer characterized by a distinct bimodal molecular weight distribution.

**Figure 22:**
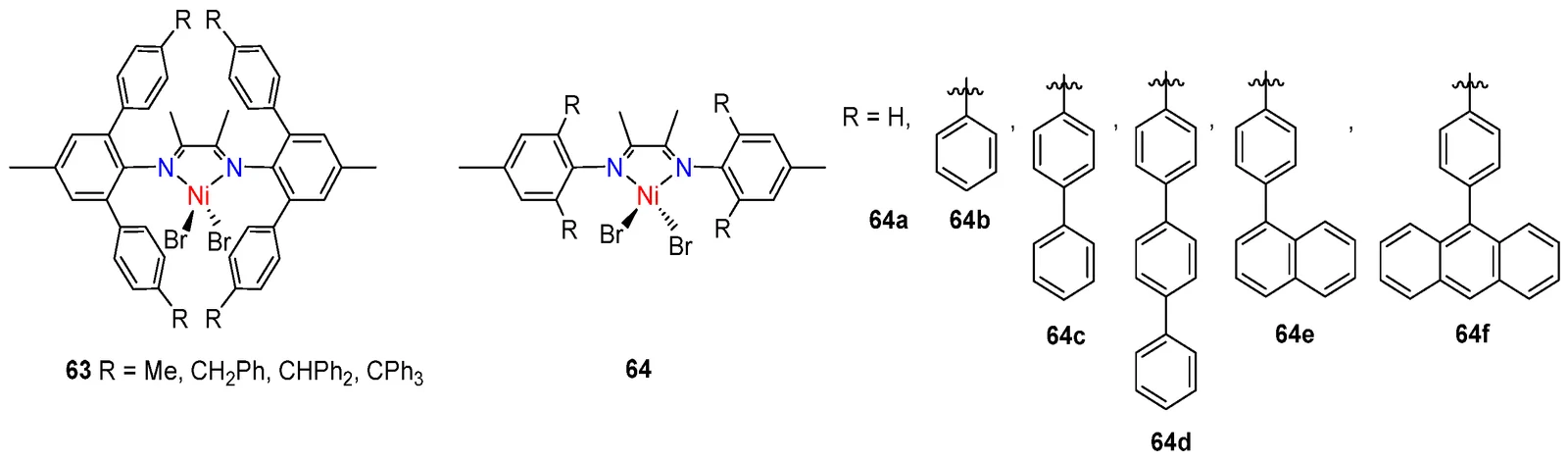
“Long arm type” *α*-diimine nickel(II) complexes **63**–**64**.

The development of “long-arm” complexes **63** and **64** (Figure 22) transcends the conventional design paradigm that relies solely on short-range steric hindrance. Complex **63**, bearing a long-arm substituent, features a unique axial environment; under atmospheric pressure, it efficiently drives polymerization by virtue of an extremely low monomer insertion barrier (43.68 kJ·mol^−1^) and affords a polymer with a number-average molecular weight (*M_n_*) as high as 3.61 × 10^5^ g·mol^−1^ [111]. Complex **64f**, which combines both lateral and axial steric confinement strategies, maintains thermal stability while boosting the catalytic activity to the 10^7^ g·mol^−1^·h^−1^ level, and successfully yields a highly linear polymer with a weight-average molecular weight (*M_w_*) of up to 5.19 × 10^6^ g·mol^−1^ and a very low branching density (2–28/1000C) [112].

**Figure 23:**
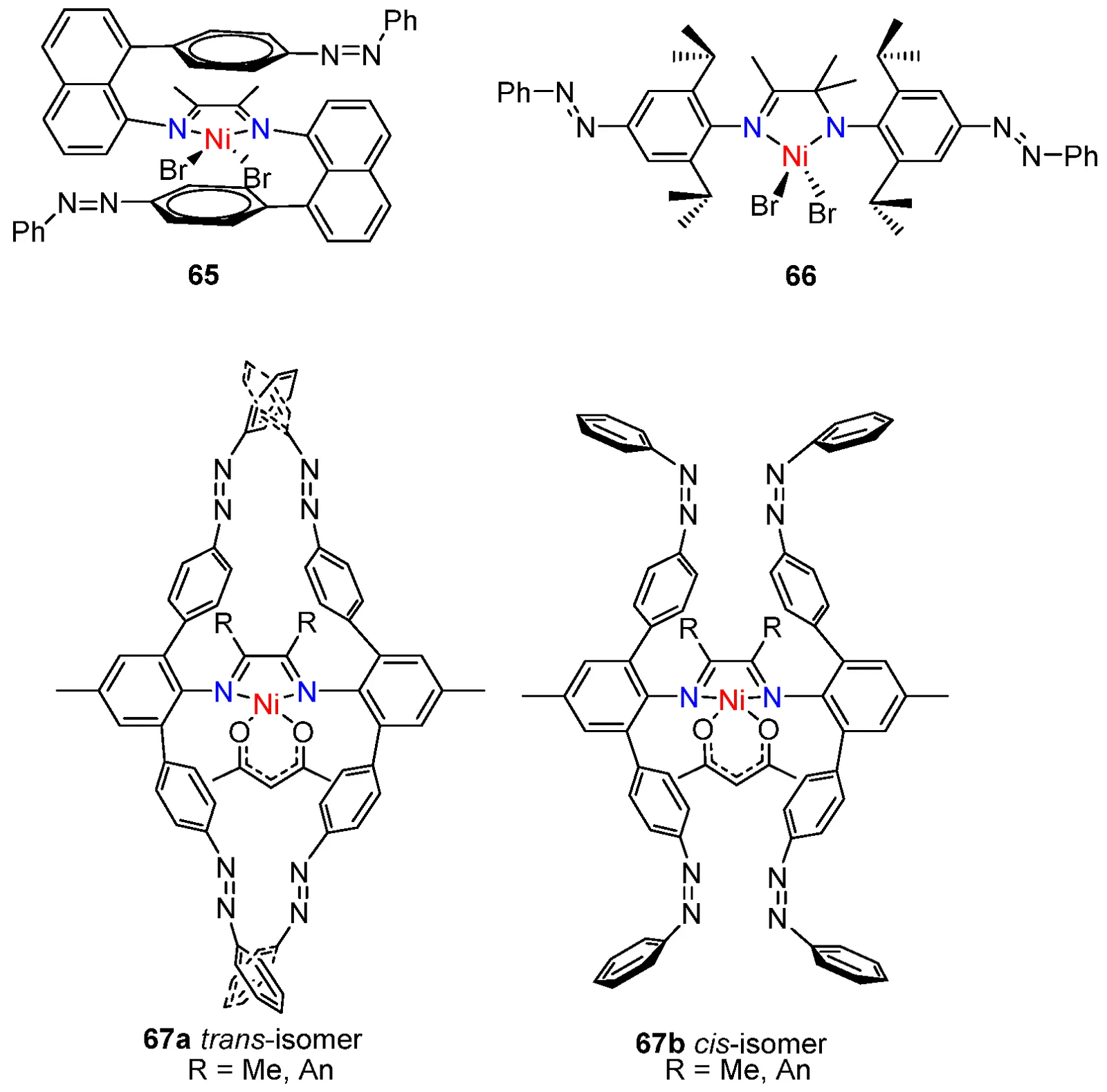
*α*-Diimine nickel(II) complexes **65**–**67** containing azobenzene unit.

In the exploration of photoisomerizable catalysts (complexes **65**–**67**, Figure 23), light serves as an intangible “switch” for modulating polymerization behavior. Inspired by the *trans*-*cis* photoisomerization of azobenzene groups upon UV irradiation, it was found that light can precisely regulate the steric crowding around the active center. For complex **65**, photoinduction leads to a decreasing trend in both catalytic activity and branching density, while the molecular weight increases [113]. In the case of complex **66**, light can trigger a *trans*-to-*cis* transformation, thereby effectively modulating the electronic effect of the ligand, which substantially enhances the activity and branching density, accompanied by a decrease in molecular weight [113]. Complex **67**, bearing four azobenzene groups, has its metal center fully shielded in the absence of light, exhibiting higher catalytic activity than under illumination, and stably affords polyethylene with higher molecular weight and lower branching density [114]. This demonstrates a highly promising prospect for utilizing external physical fields to intervene in catalytic polymerization processes in a real-time and dynamic manner.

### 3.2. Modification of the α-Diimine Backbone Structure

**Figure 24:**
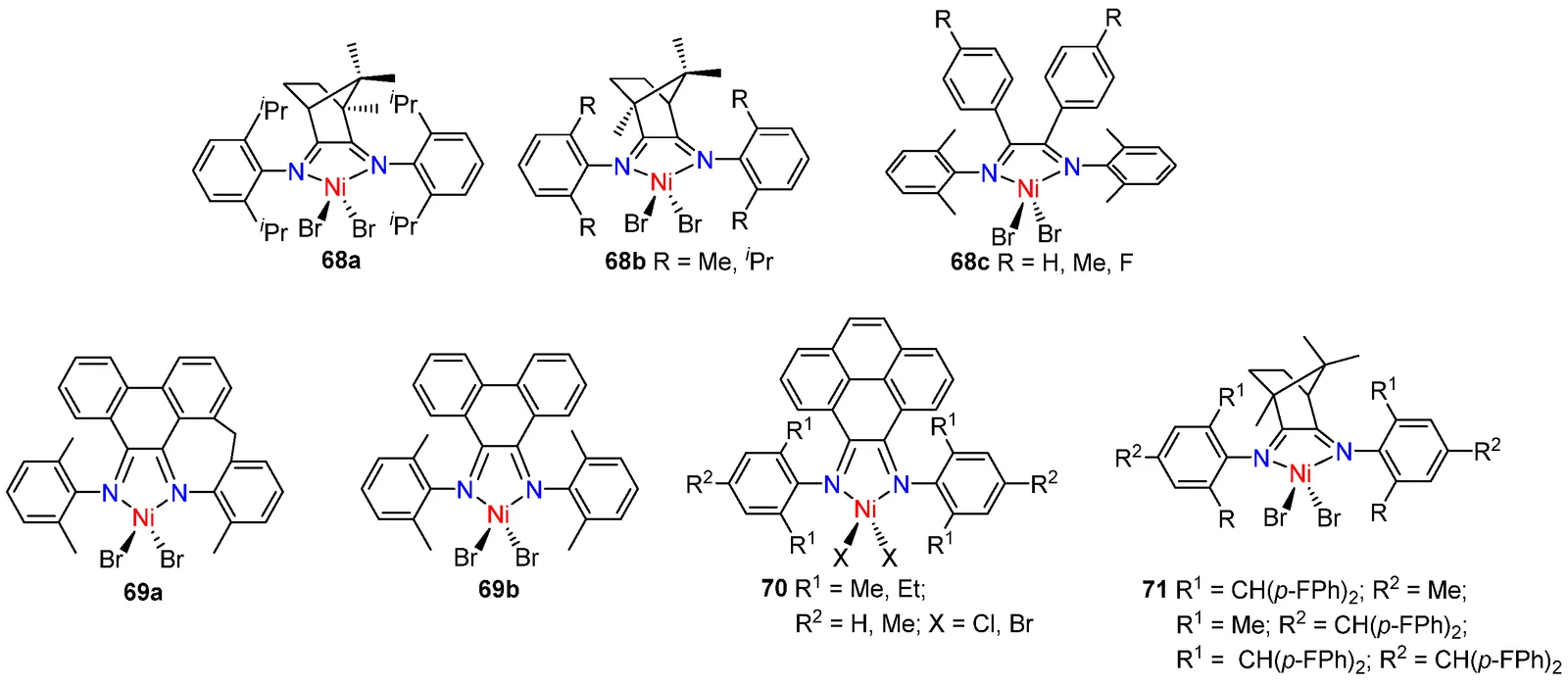
Skeleton-modified *α*-diimine nickel(II) complexes **68**–**71**.

**Figure 25:**
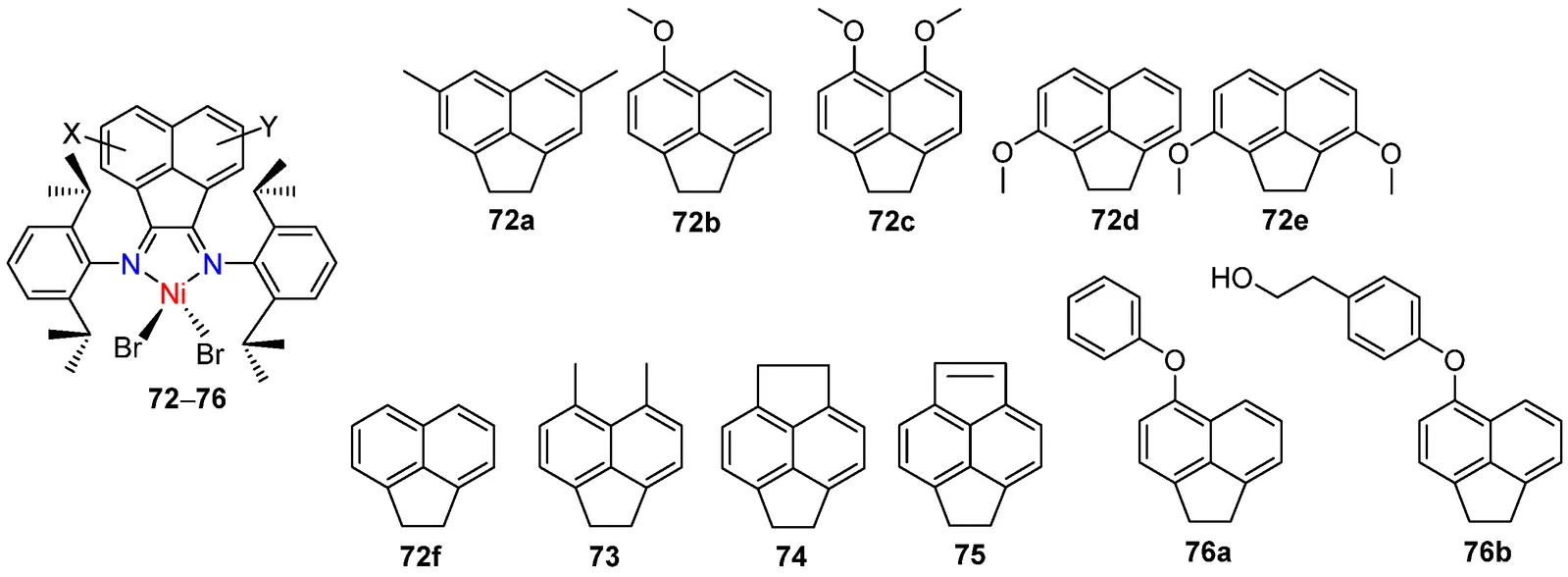
*α*-diimine nickel (II) complexes **72**–**76** with modified acenaphthene skeleton.

**Figure 26:**
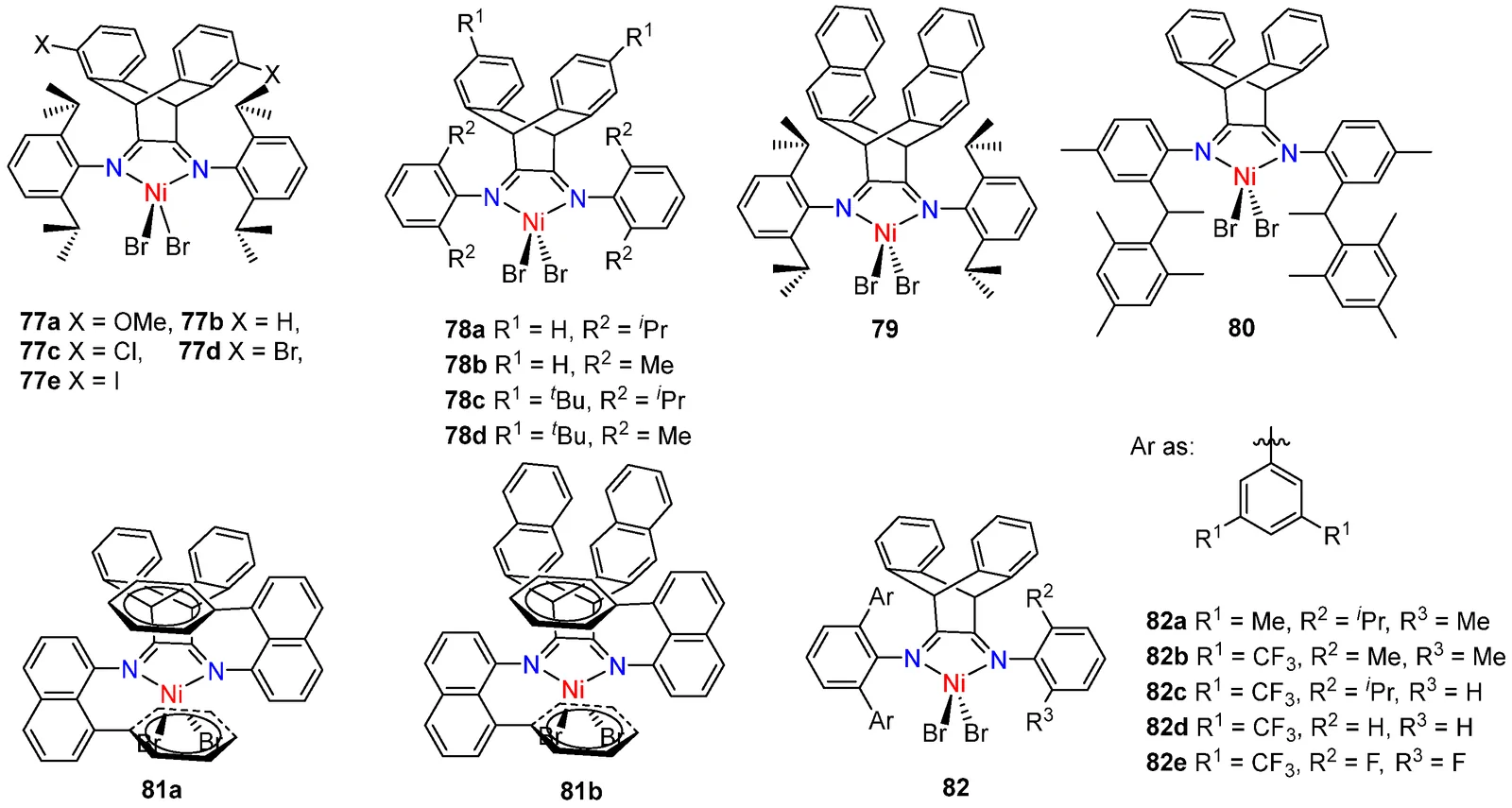
*α*-diimine nickel(II) complexes **77**–**82** containing dibenzobarrelene(dinaphthobarrelene) skeleton.

**Figure 27:**
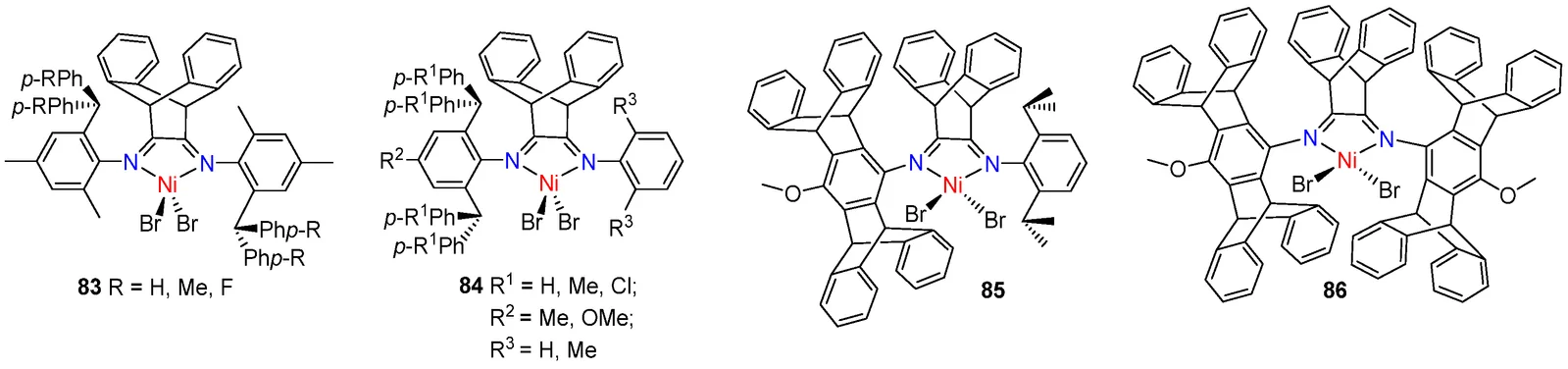
Structure strengthening *α*-diimine nickel(II) complexes **83**–**86** based on dibenzobarrelene skeleton.

**Figure 28:**
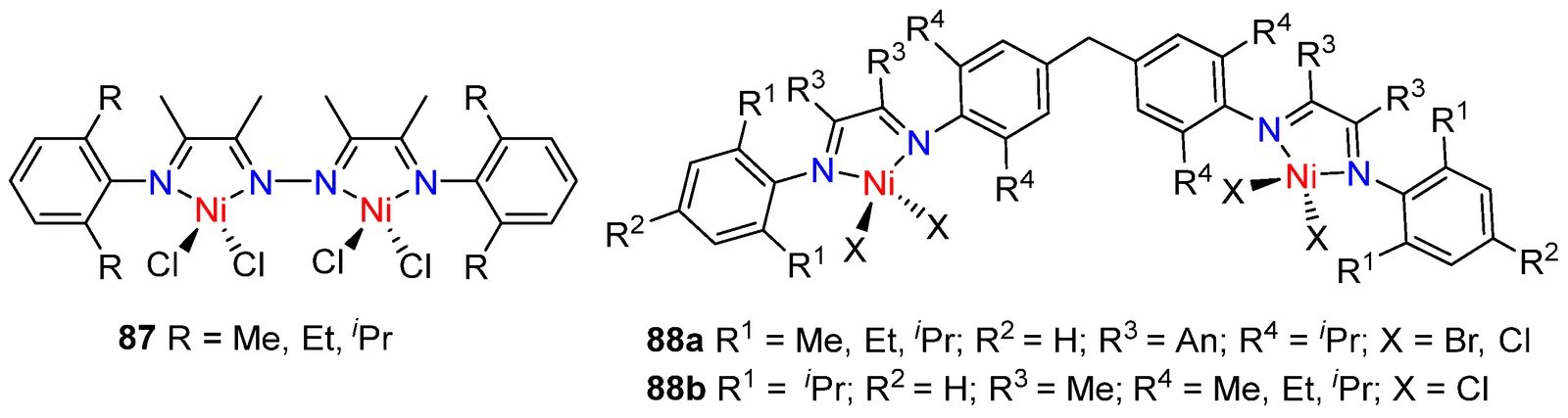
*N*,*N*-alkyl and aryl bridging binuclear *α*-diimine nickel(II) complexes **87**–**88**.

In contrast to N-aryl modification, which tunes steric and electronic effects through substituents, modification of the α-diimine backbone structure is primarily centered on changes in structural rigidity. In general, the more rigid the α-diimine backbone, the better the catalytic performance of the corresponding complex. Summarized below are α-diimine nickel(II) complexes **68**–**86** (Figure 24, Figure 25, Figure 26, Figure 27 and Figure 28), which are derived from the classic complex **1** (Figure 1) and feature different backbone structures.

#### 3.2.1. Exploration of Different α-Diimine Backbone Structures

Wu et al. introduced complexes **68a** and **68b** (Figure 24), which bear a naturally chiral camphor-derived backbone, to investigate the influence of a chiral environment on polymerization behavior. Notably, the chiral preference did not lead to any significant difference in catalytic outcomes; both complexes exhibited moderate ethylene polymerization activity of up to 5.20 × 10^5^ g·mol^−1^·h^−1^ (−10 °C, 30 min, 0.12 MPa) and produced polyethylene with high molecular weight (*M_n_* up to 3.90 × 10^5^ g·mol^−1^), narrow dispersity (PDI < 1.60), and moderate branching (40–71/1000C) [115]. Complex **68c**, which incorporates a substituted phenyl group to reinforce the backbone, shows markedly improved thermal stability, and its electron-withdrawing groups enhance the activity while significantly lowering the molecular weight of the polymer [23]. Complexes **69a** and **69b**, featuring a 9,10-phenanthrene-based backbone, both display high catalytic activity (up to 2.28 × 10^6^ g·mol^−1^·h^−1^; −15 °C, 60 min, 0.12 MPa), with **69a** capable of producing ultra-high-molecular-weight polyethylene (*M_n_* up to 2.14 × 10^6^ g·mol^−1^) at low temperature [116]. Complex **70**, built on a large conjugated 4,5-pyrenyl system, efficiently catalyzes ethylene polymerization with only a trace amount of cocatalyst (4.41 × 10^6^ g·mol^−1^·h^−1^; 40 °C, 30 min, 1 MPa), affording polyethylene with a high branching density (130/1000C) and narrow distribution (PDI < 2.5) [117]. Complex **71**, which combines a camphor-based backbone with bulky N-aryl substituents, demonstrates outstanding activity of up to 11.2 × 10^6^ g·mol^−1^·h^−1^ under high-temperature polymerization conditions (80 °C) and yields polyethylene with high molecular weight (*M_w_* up to 2.37 × 10^6^ g·mol^−1^), narrow dispersity (PDI = 1.6–2.6), and low branching density (19/1000C) [118]. The aforementioned studies further confirm that electron-withdrawing groups on the backbone play an irreplaceable and critical role in activating the catalyst [119].

#### 3.2.2. Modification and Exploration of the Acenaphthene-Based Backbone

As a typical representative of catalyst backbones, the acenaphthene-derived complex series **72** (Figure 25) exhibits excellent catalytic activity after the introduction of various substituents. The highest activity reaches 16.2 × 10^6^ g·mol^−1^·h^−1^ (20 °C, 10 min, 0.9 MPa); the resulting polyethylene has a weight-average molecular weight (*M_w_*) of 4.18 × 10^5^ g·mol^−1^ and a branching density ranging from 52 to 108/1000C [120]. In the methoxy-containing complex **72e**, strong steric repulsion generated within the structure significantly increases the molecular weight of the polymer while reducing its branching density. Complex **73**, modified with two methyl groups, not only exhibits markedly enhanced thermal stability compared to its precursor but also a greatly prolonged catalytic lifetime [121]. Complex **74**, which adopts a strategy of backbone rigidification, shows a catalytic activity at 80 °C that is up to five times that of the classic Brookhart catalyst [122,123,124]. Building on this, complex **75**, featuring a highly extended conjugated backbone, possesses a main-chain conjugated system that strongly withdraws π-electron density from the imine groups, creating a highly electron-deficient nickel center. This effect significantly accelerates the chain-walking process, ultimately producing a high-performance elastomer with a branching density as high as 135/1000C and an *M_w_* of 5.10 × 10^5^ g·mol^−1^. This elastomer exhibits outstanding mechanical properties, with a stress at break of 12.08 MPa and an elongation at break reaching 1148% [125]. Complex **76b**, which incorporates a hydroxyethylphenoxy side chain for greater practical applicability, retains an activity of 8.20 × 10^6^ g·mol^−1^·h^−1^ at 70 °C, and the hydroxyl group on the side chain provides a favorable foundation for subsequent heterogenization and industrial application [126].

#### 3.2.3. Exploration of Dibenzobarrelene- and Dinaphthobarrelene-Based Backbone Structures

Constructing intricate three-dimensional cage-like architectures on the backbone (complexes **77**–**82**, Figure 26) represents an effective physical strategy for suppressing chain transfer. For the dibenzobarrelene-bridged complex **77**, the introduction of a methoxy group locks the conformation through intramolecular hydrogen bonding, thereby markedly improving catalyst stability. The electron-withdrawing effect of halogen atoms is negatively correlated with catalytic activity; consequently, the iodine-containing complex **77e** not only displays the highest catalytic activity (5.60 × 10^5^ g·mol^−1^·h^−1^; 50 °C, 30 min, 0.12 MPa) but also yields the polymer with the highest molecular weight in this series, with a number-average molecular weight (*M_n_*) of up to 3.08 × 10^5^ g·mol^−1^ [127]. Complex **78**, which is modified with bulky 2,6-di-tert-butyl groups, maintains the stable existence of active species within its highly congested cage-like environment even at 80 °C [128]. Complex **79**, built on a dinaphthobarrelene backbone, provides stringent steric shielding that protects both the axial and rear faces of the active center in all directions, which contributes to enhanced catalytic activity and thermal stability in ethylene polymerization (highest activity: 6.96 × 10^5^ g·mol^−1^·h^−1^; 100 °C, 30 min, 0.12 MPa) and ultimately affords polyethylene with a narrow molecular weight distribution (PDI = 1.04–1.45) [129].

Through fine unilateral tuning at the orthoposition of the N-aryl ring, complex **80** significantly retards the chain-walking process, ultimately yielding nearly perfectly linear polyethylene (branching density < 1/1000C) with a weight-average molecular weight (*M_w_*) as high as 7.12 × 10^5^ g·mol^−1^ and a polydispersity index (PDI) of 1.18–1.70 [130]. Complexes **81a** and **81b**, which contain a bulky N-8-phenylnaphthyl group, exploit weak nickel–phenyl interactions within the ligand to induce rapid chain propagation and effectively suppress chain walking; both exhibit markedly enhanced thermal stability and catalytic activity (1.39 × 10^6^ g·mol^−1^·h^−1^; 80 °C, 30 min, 2 MPa) and produce semi-crystalline polymers with low branching density (<50/1000C) [131]. Complex **82**, bearing a bulky terphenyl group, features an unsymmetrical backbone structure that simultaneously ensures smooth monomer insertion and effectively blocks chain transfer. When the polymerization temperature is raised to 80 °C, the catalytic activity remains at a high level on the order of 10^6^ g·mol^−1^·h^−1^ by virtue of an intramolecular H···F hydrogen-bonding network, and the resulting polymer attains an *M_w_* of up to 1.74 × 10^6^ g·mol^−1^ [132].

Complexes **83**–**86** (Figure 27), which integrate bulky benzhydryl derivatives with a dibenzobarrelene backbone, vividly demonstrate the pronounced advantages of this structural design. The symmetric complex **83** exhibits high catalytic activity in ethylene polymerization (up to 6.98 × 10^6^ g·mol^−1^·h^−1^; 50 °C, 20 min, 0.6 MPa) and, even at 90 °C, yields semi-crystalline polyethylene with high molecular weight (*M_n_* = 7.89 × 10^5^ g·mol^−1^), a high melting point (*T_m_* = 100 °C), and low branching density (11–34/1000C) [133]. Complex **84** which bears extreme steric congestion on one side, retains an activity of 1.35 × 10^6^ g·mol^−1^·h^−1^ at 90 °C and successfully produces a high-performance elastomer with a branching density exceeding 100/1000C and a weight-average molecular weight (*M_w_*) of up to 1.27 × 10^6^ g·mol^−1^; moreover, the product maintains its highly branched character under different temperature and pressure conditions [134]. A direct comparison between complexes **85** and **86** was carried out to examine the influence of structural symmetry on catalytic performance. Both complexes are catalytically active over a broad temperature range up to 120 °C, and among them, complex **86**, which features greater steric hindrance, not only displays superior activity but also comprehensively surpasses complex **85** in terms of both polymer molecular weight and branching density [135,136].

### 3.3. Exploration of Dinuclear α-Diimine Nickel Complex Structures

In addition to investigating the influence of N-aryl substituents and ligand backbones on the catalytic performance of nickel complexes, researchers have incorporated the concept of metal-metal cooperativity into catalyst design and have conducted extensive explorations in late-transition-metal-catalyzed olefin polymerization [137,138]. In bimetallic complexes, the intermetallic distance is a critical factor that governs the metal-metal cooperativity during ethylene polymerization and exerts a pronounced influence on both the polymer microstructure and the chain-walking process.

Dinuclear nickel complex **87** (Figure 28), which is bridged by a diazine unit, catalyzes ethylene oligomerization with an activity of up to 1.12 × 10^6^ g·mol^−1^·h^−1^ (30 °C, 30 min, 0.13 MPa) [139]. Complex **88a**, constructed by tightly bridging two acenaphthene-based catalytic centers through a methylene linker, displays high catalytic activity in ethylene polymerization (7.86 × 10^6^ g·mol^−1^·h^−1^; 50 °C, 30 min, 1 MPa) and yields polyethylene with a high melting point (*T_m_* = 130.9 °C) and a high branching density (151/1000C) [140]. More revealingly, the activity of this dinuclear structure far exceeds that of the mononuclear complex obtained by its dissection, offering rare and compelling evidence for a “positive bimetallic synergistic effect”. The subsequent complex **88b** system further discloses that the close proximity of the two active centers can substantially alter the electronic arrangement in the environment surrounding the metal centers [141].

**Figure 29:**
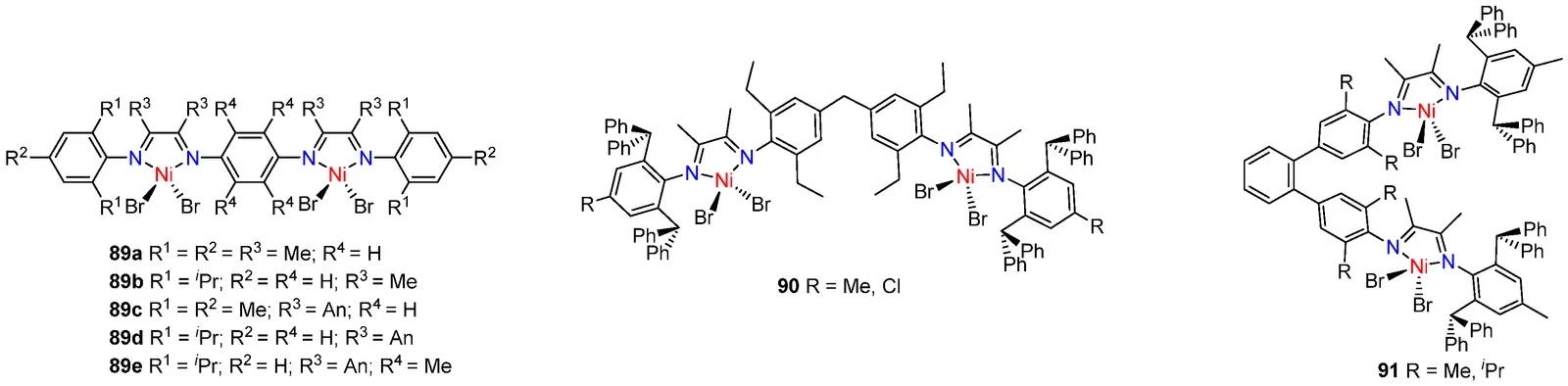
Aryl bridging binuclear *α*-diimine nickel(II) complexes **89**–**91**.

Among the dinuclear catalyst systems bridged by a 1,4-phenylene unit, complex **89d** (Figure 29), which contains isopropyl substituents, exhibits the highest catalytic activity, reaching 1.07 × 10^6^ g·mol^−1^·h^−1^ (42 °C, 20 min, 0.15 MPa), and produces polyethylene with a broad molecular weight distribution [142]. Complex **90**, in which a bulky benzhydryl group is further introduced onto the dinuclear backbone, displays superior activity relative to its mononuclear counterpart, with an optimal activity of approximately 1.51 × 10^6^ g·mol^−1^·h^−1^ (30 °C, 30 min, 0.6 MPa), and yields polyethylene with high molecular weight (*M_n_* up to 4.46 × 10^5^ g·mol^−1^), high branching density (79–92/1000C), and outstanding mechanical properties (strain recovery, SR, up to 84%) [143]. Complex **91**, featuring an *o*-phenylene linker, not only significantly outperforms the corresponding mononuclear analogue in activity, but also enables the synthesis of customized elastomeric materials with strain recovery tunable over the range of 29–86% through precise modulation of reaction conditions [144].

**Figure 30:**
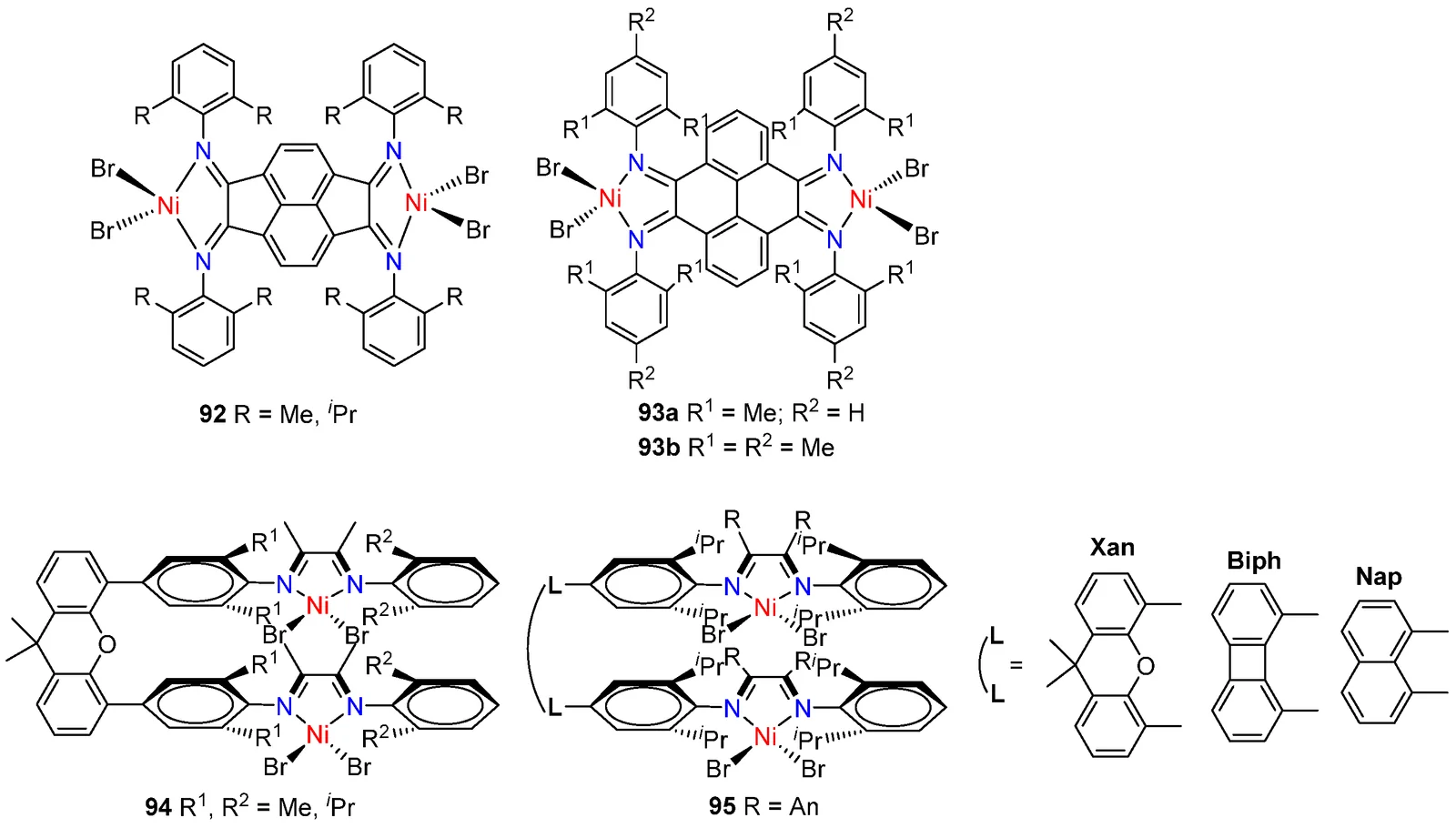
Binuclear *α*-diimine nickel(II) complexes **92**–**95** containing conjugated skeleton.

When the bridging framework is highly conjugated (complex **92**, Figure 30), the catalyst not only achieves an activity of 1.05 × 10^6^ g·mol^−1^·h^−1^ under ambient conditions (22 °C, 0.5 MPa, 30 min), but the bulky borate anions added to the polymerization system can also effectively stabilize the active centers at elevated temperatures [145]. Dinuclear complexes **93a** and **93b**, designed and synthesized on the basis of a bulky 4,5,9,10-tetrapyrenyl framework, display comparable activities; the former exhibits an activity of 1.50 × 10^6^ g·mol^−1^·h^−1^ (30 °C, 30 min, 1 MPa) and yields linear polyethylene resin with low branching density (7–14/1000C) and a high melting point (132 °C) [146,147]. The key discovery concerning the mechanism by which bimetallic synergistic effects suppress chain transfer originates from dinuclear complexes **94** and **95**, which are bridged by large conjugated structures such as xanthene, naphthalene, and biphenyl. Taking complex **88** as an example, its catalytic activity reaches up to 5.00 × 10^6^ g·mol^−1^·h^−1^ (20 °C, 30 min, 0.9 MPa), producing polyethylene with a maximum molecular weight (*M_n_*) of 2.39 × 10^5^ g·mol^−1^. Notably, under identical conditions, its mononuclear analogue displays an activity of only 4.60 × 10^6^ g·mol^−1^·h^−1^ and an *M_n_* of 1.49 × 10^5^ g·mol^−1^; when the reaction temperature is raised to 80 °C, both the catalytic activity and the product molecular weight of complex **96** reach nearly twice those of the corresponding mononuclear analogue. Thorough physicochemical analyses confirm that this phenomenon is attributed to a strong Ni-Ni through-space cooperativity arising between the two adjacent nickel centers; this intermetallic electronic interaction can effectively suppress β-hydride elimination, thereby significantly retarding the chain-walking process [18]. The similarly excellent results exhibited by complex **95** further provide solid experimental evidence for the theory of dinuclear cooperative catalysis with late transition metals [148].

## 4. Summary and Outlook

Brookhart-type α-diimine nickel(II) complexes have been extensively investigated owing to their unique chain-walking capability. The polymerization activity of these catalysts and the microstructural features of the resulting polymers can be finely regulated by tailoring the ligand structure and polymerization conditions. This review has summarized the main achievements in this field. The chain-walking mechanism and the principles governing the formation of various branched polymer architectures have been elucidated. Systematic ligand-modification strategies—including steric, electronic, configurational, and bimetallic effects—have been demonstrated to alter the electron density at the metal center and the steric shielding at the axial coordination sites, thereby effectively intervening in chain propagation and chain transfer. These advances have provided viable routes to high-activity, thermally robust catalytic systems and have enabled the synthesis of high-value-added polyethylene topologies, such as polyethylene elastomers, ultra-high-molecular-weight polyethylene, and low-molecular-weight branched polyethylene, all from ethylene as the sole monomer. Of particular note, hyperbranched polyethylene with thermoplastic elastomer properties holds great promise as a substitute for traditional elastic ethylene copolymers.

Despite these remarkable advances, the rational design of advanced catalytic systems still faces several critical challenges, and addressing these will present significant opportunities for future investigators. First, improving catalyst stability at elevated temperatures without compromising polymerization activity remains a primary hurdle. Future designs must explore more robust ligand frameworks to prevent catalyst deactivation and chain transfer under harsh industrial conditions. Second, achieving precise branching control—specifically regulating branch type, length, and distribution along the polymer backbone—is essential for tailoring polymer architectures to specific needs. Third, deeper insights into structure–property relationships are urgently required to accurately map how specific microstructural topological features dictate macroscopic mechanical and rheological properties, thereby guiding the customized design of novel resins. Furthermore, evaluating the industrial scalability of these catalysts is imperative; bridging the gap between laboratory-scale synthesis and large-scale, cost-effective production will be a critical next step. Finally, the integration of emerging computational approaches, such as density functional theory (DFT) combined with machine learning (ML), offers a powerful toolset. These computational methods can accelerate the discovery of next-generation α-diimine nickel(II) complexes by predicting catalyst behavior and simulating polymer microstructures prior to empirical synthesis.

By overcoming these challenges, it is anticipated that the quality of branched polyethylene resins can be precisely modulated, facilitating the large-scale application of advanced polyethylene elastomers in high-demand industries, including microelectronic packaging, vehicle manufacturing, and footwear production.

## Figures and Tables

**Table 1:** Mechanical properties of selected PEE samples obtained under varying conditions using nickel precatalysts **35**–**38**.

Entry	Cat.	Cocat.	T (°C) *^a^*	Act. (10^6^) *^b^*	M*_w_* (10^5^) *^c^*	T*_m_* (°C) *^d^*	BD *^e^*	σ*_b_* (MPa) *^f^*	ɛ (%) *^g^*	SR (%) *^h^*	Ref.
1	**35**	EASC	20	8.41	1.64	124.1	-	-	-	-	[77]
2	**36**	MAO	30	10.56	5.90	113.3	63	-	-	-	[78]
3	**37**	MMAO	70	0.83	9.05	-	89	-	-	-	[79]
4	**38a**	Et_2_AlCl	30	13.92	0.07	25	68	-	-	-	[80]
5	**38b**	Et_2_AlCl	30	10.9	2.9	57.9	57	4.2	1526	40	[81]

*^a^* Reaction temperature, *^b^* catalytic activity, unit g mol^−1^ h−^1^, *^c^* polymer molecular weight (M*_w_* or M*_n_*), unit: g mol^−1^, *^d^* polymer melting temperature, *^e^* branching density: per 1000 carbon, *^f^* Stress at break, *^g^* Strain at break, *^h^* strain recovery.

**Table 2:** Mechanical properties of selected PEE samples obtained under varying conditions using nickel precatalysts **39**–**44**.

Entry	Cat.	Cocat.	T (°C) *^a^*	Act. (10^6^) *^b^*	M*_w_* (10^5^) *^c^*	T*_m_* (°C) *^d^*	BD *^e^*	σ*_b_* (MPa) *^f^*	ɛ (%) *^g^*	SR (%) *^h^*	Ref.
1	**39a**	EASC	50	12.2	0.60	102.7	57	12.2	787	55	[82]
2	**39b**	Et_2_AlCl	55	8.8	1.52	114.7	62	14.1	1635	62	[83]
3	**40**	Me_2_AlCl	30	11.7	2.60	109.3	121	11.0	1044	46	[84]
4	**41a**	Et_2_AlCl	30	19.9	7.46	77.8	101	11.1	700	68	[86]
5	**41b**	EASC	40	21.4	6.41	58.7	90	15.3	482	-	[85]
6	**42**	Me_2_AlCl	30	7.4	1.57	81.7	134	8.6	1797	40	[87]
7	**43**	EASC	40	10.3	1.90	58.0	87	3.2	1589	52	[88]
8	**44**	Et_2_AlCl	30	7.5	3.20	79.1	56	8.9	1251	42	[89]

*^a^* Reaction temperature, *^b^* catalytic activity, unit g mol^−1^ h−^1^, *^c^* polymer molecular weight (M*_w_* or M*_n_*), unit: g mol^−1^, *^d^* polymer melting temperature, *^e^* branching density: per 1000 carbon, *^f^* Stress at break, *^g^* Strain at break, *^h^* strain recovery.

## Data Availability

No new data were created or analyzed in this study. Data sharing is not applicable to this article.
